# Anger and disgust shape judgments of social sanctions across cultures, especially in high individual autonomy societies

**DOI:** 10.1038/s41598-024-55815-x

**Published:** 2024-03-07

**Authors:** Per A. Andersson, Irina Vartanova, Daniel Västfjäll, Gustav Tinghög, Pontus Strimling, Junhui Wu, Isabela Hazin, Charity S. Akotia, Alisher Aldashev, Giulia Andrighetto, Adote Anum, Gizem Arikan, Fatemeh Bagherian, Davide Barrera, Dana Basnight-Brown, Birzhan Batkeyev, Elizaveta Berezina, Marie Björnstjerna, Paweł Boski, Inna Bovina, Bui Thi Thu Huyen, Đorđe Čekrlija, Hoon-Seok Choi, Carlos C. Contreras-Ibáñez, Rui Costa-Lopes, Mícheál de Barra, Piyanjali de Zoysa, Angela R. Dorrough, Nikolay Dvoryanchikov, Jan B. Engelmann, Hyun Euh, Xia Fang, Susann Fiedler, Olivia A. Foster-Gimbel, Márta Fülöp, Ragna B. Gardarsdottir, C. M. Hew D. Gill, Andreas Glöckner, Sylvie Graf, Ani Grigoryan, Vladimir Gritskov, Katarzyna Growiec, Peter Halama, Andree Hartanto, Tim Hopthrow, Martina Hřebíčková, Dzintra Iliško, Hirotaka Imada, Hansika Kapoor, Kerry Kawakami, Narine Khachatryan, Natalia Kharchenko, Toko Kiyonari, Michal Kohút, Lisa M. Leslie, Yang Li, Norman P. Li, Zhuo Li, Kadi Liik, Angela T. Maitner, Bernardo Manhique, Harry Manley, Imed Medhioub, Sari Mentser, Pegah Nejat, Orlando Nipassa, Ravit Nussinson, Nneoma G. Onyedire, Ike E. Onyishi, Penny Panagiotopoulou, Lorena R. Perez-Floriano, Minna Persson, Anna-Maija Pirttilä-Backman, Marianna Pogosyan, Jana Raver, Ricardo Borges Rodrigues, Sara Romanò, Pedro P. Romero, Inari Sakki, Alvaro San Martin, Sara Sherbaji, Hiroshi Shimizu, Brent Simpson, Erna Szabo, Kosuke Takemura, Maria Luisa Mendes Teixeira, Napoj Thanomkul, Habib Tiliouine, Giovanni A. Travaglino, Yannis Tsirbas, Sita Widodo, Rizqy Zein, Lina Zirganou-Kazolea, Kimmo Eriksson

**Affiliations:** 1 Department of Behavioral Sciences and Learning, Linköping University, 581 83 Linköping, Sweden; 2 JEDILab, Division of Economics, Department of Management and Engineering, Linköping University, Linköping, Sweden; 3 Institute for Futures Studies, Box 591, 101 31 Stockholm, Sweden; 4 JEDILab, Department of Health, Medicine and Caring Sciences, Linkoping University, Linkoping, Sweden; 5 CAS Key Laboratory of Behavioral Science, Institute of Psychology, Chinese Academy of Sciences, Lincui Road 16, Chaoyang District, Beijing, 100101 China; 6 Department of Psychology, University of Chinese Academy of Sciences, Beijing, China; 7 Department of Women’s and Children’s Health, University of Uppsala, Uppsala, Sweden; 8 Department of Psychology, University of Ghana, Legon, P.O. Box LG 84, Accra, Ghana; 9 International School of Economics, Kazakh-British Technical University, 59 Tole Bi Street, 050000 Almaty, Kazakhstan; 10 Mälardalen University, 721 23 Västerås, Sweden; 11 Institute of Cognitive Sciences and Technologies, National Research Council of Italy, 00185 Rome, Italy; 12 Department of Political Science, Trinity College Dublin, 2-3 College Green, Dublin 2, Ireland; 13 Faculty of Education and Psychology, Shahid Beheshti University, Tehran, 1983969411 Iran; 14 University of Turin and Collegio Carlo Alberto, Lungo Dora Siena 100, 10124 Turin, Italy; 15 School of Humanities and Social Sciences, United States International University Africa, Box 14634 00800, Nairobi, Kenya; 16 Sunway University, No. 5, Jalan Universiti, Bandar Sunway, 47500 Petaling Jaya, Selangor Darul Ehsan Malaysia; 17 SWPS University, Chodakowska 19-31, 03-815 Warsaw, Poland; 18 Moscow State University of Psychology and Education, Sretenka Str., 29, 127051 Moscow, Russia; 19 Hanoi National University of Education, 136 Xuan Thuy Street, Cau Giay District, Hanoi, Vietnam; 20 Faculty of Philosophy, University of Banja Luka, Vojvode Petra Bojovića 1A, 78000 Banja Luka, Bosnia and Herzegovina; 21 Department of Psychology, Sungkyunkwan University, 25-2, Sungkyunkwan-Ro, Jongno-Gu, Seoul, 03063 Republic of Korea; 22 Departamento de Sociología, Universidad Autónoma Metropolitana-Unidad Iztapalapa, Av. Rafael Atlixco 186, Col. Vicentina, 09340 Ciudad de México, México; 23 Instituto de Ciências Sociais, Universidade de Lisboa, Av. Prof. Anibal de Bettencourt, 9, 1600-189 Lisboa, Portugal; 24 Center for Culture and Evolution, Brunel University London, Uxbridge, UB8 3PH UK; 25 Faculty of Medicine, University of Colombo, Kynsey Road, Colombo 8, Sri Lanka; 26 Department of Psychology, University of Cologne, Richard-Strauss-Str. 2, 50931 Cologne, Germany; 27 Center for Research in Experimental Economics and Political Decision Making (CREED), Amsterdam School of Economics, University of Amsterdam, P.O. Box 15867, 1001 NJ Amsterdam, The Netherlands; 28 Gies College of Business, University of Illinois at Urbana-Champaign, 1206 S 6Th St., Champaign, IL 61820 USA; 29 Department of Psychology and Behavioral Sciences, Zhejiang University, 148 Tianmushan Road, Hangzhou, Zhejiang China; 30 Vienna University of Economics and Business, Welthandelsplatz 1, 1020 Vienna, Austria; 31 Stern School of Business, New York University, 40 West 4Th Street, Tisch Hall, Suite 700, New York, NY 10012 USA; 32 HUN-REN Institute of Cognitive Neuroscience and Psychology, Research Centre of Natural Sciences, Budapest, Hungary; 33 Károli Gáspár University of the Reformed Church, Bécsi Út 324, Budapest, 1034 Hungary; 34 Faculty of Psychology, University of Iceland, Nyi Gardur, Saemundargata 12, IS-102 Reykjavík, Iceland; 35 Universal College Bangladesh, Dhaka, Bangladesh; 36 Max Planck Institute for Research on Collective Goods, Kurt-Schumacher-Str. 10, 53113 Bonn, Germany; 37 Institute of Psychology, Czech Academy of Sciences, Veveří 97, 602 00 Brno, Czech Republic; 38 Department of Personality Psychology, Yerevan State University, Alex Manoogian 1, 0025 Yerevan, Armenia; 39 Saint Petersburg State University, 7-9 Universitetskaya Emb., St Petersburg, 199034 Russia; 40 Center for Social and Psychological Sciences, Slovak Academy of Sciences, Dubravska Cesta 9, 841 04 Bratislava, Slovakia; 41 School of Social Sciences, Singapore Management University, 90 Stamford Road, Singapore, 178903 Singapore; 42 School of Psychology, University of Kent, Canterbury, CT2 7NP UK; 43 Daugavpils University, Latvia, Parades Street 1, Room 432, Daugvapils, 5400 Latvia; 44 Department of Psychology, Royal Holloway, University of London, Egham, TW20 0EX UK; 45 Department of Psychology, Monk Prayogshala, 4114, C Wing, Oberoi Garden Estates, Off Saki Vihar Road, Andheri East, Mumbai, Maharashtra 400072 India; 46 Department of Psychology, York University, 4700 Keele Street, Toronto, ON Canada; 47 Kyiv International Institute of Sociology, Voloska Str., 8/5, Build. 4, Kyiv, 04070 Ukraine; 48 Aoyama Gakuin University, 5-10-1, Fuchinobe, Chuo-Ku, Sagamihara-City, Kanagawa 252-5258 Japan; 49 Faculty of Philosophy and Arts, University of Trnava, Hornopotočná 23, 918 43 Trnava, Slovakia; 50 Melbourne School of Psychological Science, University of Melbourne, 1116 Redmond Barry Building, Melbourne, VIC 3010 Australia; 51 Department of Psychology, University of Western Ontario, 1151 Richmond St, London, ON N6A 5C2 Canada; 52 School of Natural Sciences and Health, Tallinn University, Narva Rd 25, 10120 Tallinn, Estonia; 53 Department of Psychology, American University of Sharjah, PO Box 26666, Sharjah, United Arab Emirates; 54 Faculty of Arts and Social Sciences, Department of Sociology, Eduardo Mondlane University, Av. Julius Nyerere, 3453, Main Campus, Maputo, Mozambique; 55 Faculty of Behavioral Sciences, Education, & Languages, HELP University Subang 2, Subang Jaya, Malaysia; 56 Faculty of Psychology, Chulalongkorn University, 254 Phayathai Road, Pathumwan, Bangkok, 10330 Thailand; 57 Department of Finance, Imam Mohammad Ibn Saud Islamic University (IMSIU), P.O. Box 5701, Riyadh, Saudi Arabia; 58 Department of Education and Psychology, The Open University of Israel, 1 University Road, 4353701 Raanana, Israel; 59 Institute of Information Processing and Decision Making, University of Haifa, Abba Khoushy Ave 199, 3498838 Haifa, Israel; 60 Department of Psychology, University of Nigeria, Nsukka, 41000 Nigeria; 61 Department of Education and Social Work, University of Patras, 26500 Rion, Patras Greece; 62 Facultad de Economía y Empresa, Universidad Diego Portales, Av. Sta. Clara 797, Huechuraba, Región Metropolitana Chile; 63 Faculty of Social Sciences, Social Psychology, University of Helsinki, PO Box 54 (Unioninkatu 37), 00014 Helsinki, Finland; 64 Leadership and Management, Amsterdam Business School, University of Amsterdam, PO Box 15953, 1001 NB Amsterdam, The Netherlands; 65 Queen’s University, Goodes Hall, Kingston, ON K7L 3N6 Canada; 66 Instituto Universitário de Lisboa ISCTE-IUL, CIS, Avenida das Forças Armadas, 1649-026 Lisbon, Portugal; 67 Department of Culture, Politics and Society, University of Turin, 10135 Turin, Italy; 68 Experimental and Computational Economics Lab (ECEL), School of Economics, Universidad San Francisco de Quito USFQ, Diego de Robles y Pampite, Quito, Ecuador; 69 Faculty of Social Sciences, Social Psychology, University of Helsinki, PO Box 42 (Unioninkatu 33), 00014 Helsinki, Finland; 70 IESE Business School, Camino del Cerro del Águila, 3, 28023 Madrid, Spain; 71 Department of International Studies, American University of Sharjah, PO Box 26666, Sharjah, United Arab Emirates; 72 Department of Anthropology, University College London, Gower Street, London, WC1E 6BT UK; 73 Kwansei Gakuin University, 1-155 Uegahara 1Bancho, Nishinomiya, Hyogo 662-8501 Japan; 74 Department of Sociology, University of South Carolina, Columbia, SC 29208 USA; 75 Department of International Management, Johannes Kepler University, Altenberger Str. 69, 4040 Linz, Austria; 76 Faculty of Economics, Shiga University, 1-1-1 Banba, Hikone, Shiga 522-8522 Japan; 77 Mackenzie Presbyterian University, Business Administration Postgraduate Program, Consolação St, 930, São Paulo, CEP 01302-000 Brazil; 78 Labo-PECS, Faculty of Social Sciences, Université d’Oran 2, 31000 Oran, Algeria; 79 Department of Law and Criminology, Institute for the Study of Power, Crime, and Society, Royal Holloway, University of London, Egham, TW20 0EX UK; 80 Department of Political Science and Public Administration, University of Athens, 6 Themistokleous Street, 10678 Athens, Greece; 81 Department of Psychology, Universitas Airlangga, Kampus B Unair Jalan Airlangga 4-6, Surabaya, 60286 Indonesia; 82 The Institute for Analytical Sociology, Linköping University, Linköping, Sweden; 83 Institute of Psychology, University of Greifswald, Greifswald, Germany

**Keywords:** Psychology, Human behaviour

## Abstract

When someone violates a social norm, others may think that some sanction would be appropriate. We examine how the experience of emotions like anger and disgust relate to the judged appropriateness of sanctions, in a pre-registered analysis of data from a large-scale study in 56 societies. Across the world, we find that individuals who experience anger and disgust over a norm violation are more likely to endorse confrontation, ostracism and, to a smaller extent, gossip. Moreover, we find that the experience of anger is consistently the strongest predictor of judgments of confrontation, compared to other emotions. Although the link between state-based emotions and judgments may seem universal, its strength varies across countries. Aligned with theoretical predictions, this link is stronger in societies, and among individuals, that place higher value on individual autonomy. Thus, autonomy values may increase the role that emotions play in guiding judgments of social sanctions.

## Introduction

Social norms are ubiquitous features of human societies^1^. Central to understanding norms are judgments of what responses are appropriate when a norm is violated. Informal social sanctions directed at the norm violator is one such response, which is common across ages and cultures^2,3^. People’s judgments of the appropriateness of informal sanctions influence their use of sanctions, and are therefore key to knowing when the norms and practices of a society will change versus be enforced^4^. How appropriate a given sanction is perceived to be depends on many non-individual factors, such as the kind of sanction, the severity of the norm violation, and culture^5–8^, but it also depends on the observer’s emotions elicited by the norm violation. Emotions have deep roots to behavior, for example, disgust is thought to serve the purpose of getting people to avoid pathogens, but it also leads to avoiding people who elicit disgust^9,10^. Anger, in turn, has been hypothesized to promote the bargaining position of the angry person in influencing others to bend towards their will^11^, as well as being part of a threat management system^12^. To which degree such relations between emotions and specific behavior are biologically rooted or culturally learned is still debated^13^. While this present study investigates relations between emotions and behavior, we do not focus on their biological or cultural origins, but rather statistical similarities and differences in the present.

The emotional experiences of anger and disgust are important as they often precede and predict behaviors such as avoiding and punishing people who break norms. A wide range of studies have investigated anger and disgust as reactions to norm violations. Such studies include norm violations where the violation causes harm^14,15^, is harmless but disgust-eliciting^16,17^, or is a relatively harmless everyday uncivil behavior^18^. Whether the norm being violated should be considered moral or merely conventional in these studies depend partly on one’s culture and view of morality^19,20^. The relation between emotions in response to norm violations and specifically judgments regarding sanctioning the violator have also been investigated in a wide range of studies^16,21–25^. Common among the mentioned studies of norm violations and emotions, is that specifically anger and disgust have been identified as correlates of judgments regarding sanctioning the norm violator. However, the universality of these findings cannot be taken for granted, because studies in this area have typically been conducted in WEIRD countries (Western, Educated, Industrialized, Rich and Democratic) whose populations are not necessarily representative of the global population^26^.

There is a long-standing debate regarding the cultural universality of emotions and their expressions^13,27–29^. For example, some research questions whether the facial expression associated with disgust is universally understood^30^, other research notes that the eliciting context and behavioral consequences of disgust may vary^31^. However, that people at times experience emotions such as what can broadly be defined as disgust appears to be universal^31^. More recent accounts of emotion stress that while there might be core similarities in emotions across cultural groups, emotions are also culturally constructed^13^. This means that how a specific emotion is defined, recognized and expressed in behavior may vary due to cultural differences. It is therefore possible, but by no means self-evident, that the link between the emotions of anger and disgust and the desire for social sanctions is shared across countries. Here, we sought to investigate this link, focusing on individual-level state-based emotions in response to norm violations. Thus, we propose our first hypothesis.

**Hypothesis 1** Across societies, feelings of anger and disgust over a norm violation are related to sanctions being judged as more appropriate.

Note that this hypothesis does not differentiate between anger and disgust. Indeed, some scholars argue that anger and disgust are not meaningfully distinct emotions in judgments of norm violations^32,33^. However, other scholars argue that anger and disgust are predictive of different kinds of behavior, with the former predicting approaching behavior^34^ and the latter predicting avoidance behavior^35^. Looking specifically at judgments of social sanctions, we build on recent research and distinguish between three kinds of social sanctions: confrontation, ostracism, and gossip^8,36^. We chose these sanctions for this present study because of their mentioned connection to emotion in prior literature, their occurrence in everyday life^36^, and because they are separable into direct and indirect punishment^37^. Studies show associations between preferences for such sanctions and felt emotions, with anger as a stronger predictor of confrontation, an approaching behavior, while disgust has been a stronger predictor of ostracism or avoidance^23,25,38,39^. The extent to which these distinctions are universal is still unknown. To properly understand the link between emotions and social sanctions, we will address this issue of specificity by examining the extent to which ratings of confrontation, ostracism, and gossip are linked to distinct emotions about the norm violation.

Hypothesis 1 predicts a universal effect of anger and disgust emotions on judgments of sanctions, in the sense that the relation would exist across cultures. Even if emotions impact such judgments, there may still be systematic differences in the strength of this effect. Emotions may play an even larger role in some societies, and for some individuals, than in others since judgments about appropriate behavior are influenced by other factors as well. As mentioned earlier, cultural traditions vary between countries, and can be an important source of judgments for what is considered appropriate behavior. As another example, religion influences how people make judgments of right and wrong, by making explicit claims of this kind^40,41^. The influence that such institutions have on judgments is likely to vary across cultures, particularly those that differ in individual autonomy. Low-autonomy cultures tend to put a higher value on respect for tradition, religion, social order, and obedience^42^. In other words, people in low-autonomy cultures should be more influenced by sources external to themselves for guidance, such as by tradition and religion. These external sources of guidance for how to act become less important in cultures that more strongly promote individual autonomy. As a consequence of relying less on external sources of guidance, we propose that individuals would instead be more guided by internal factors, such as their own emotions and intuitions. For example, individual autonomy should lead to greater reliance on feelings of anger and disgust when judging how appropriate a sanction is. Thus, we propose our second hypothesis, which is novel:

**Hypothesis 2** Higher autonomy values lead to a stronger relation between feelings of anger and disgust over a norm violation and judgments of sanctions as more appropriate.

Note that Hypothesis 2 may be examined both at the country level and the individual level. We test our two hypotheses using previously unpublished data on emotions elicited in a study on norm violations among 17,774 participants in 56 countries, spread across all continents except Antarctica, collected in the International Study of Metanorms^8^. The norm violations we use are relatively mild violations of conventions, but they have been rated as inappropriate across all measured cultures. Prior to data analysis, we pre-registered Hypotheses 1 and 2 and the primary analyses through the Open Science Framework (https://osf.io/48cyn). To preview our results, both hypotheses are supported in these analyses. Thus, judgments of social sanctions are consistently found to be more positive among people who feel anger and disgust over the norm violation, and this link is stronger in high-autonomy cultures. Moreover, we find that the experience of anger is linked more strongly to judgments of confrontation. Neither emotion has a strong impact on judgments of gossip. Judgments of ostracism are predicted by both anger and disgust. Taken together, these findings provide a clear view of the role that emotions play in how people across the globe think about the appropriateness of specific social sanctions—and how the role of emotions varies across cultures.

## Results

Our results concern participants’ ratings of the appropriateness of various sanctions, using a six-point scale ranging from extremely inappropriate to extremely appropriate. These ratings were made in response to norm violations. In terms of procedure, participants first viewed a norm violation scenario (e.g., singing in a library) and then rated the appropriateness of the norm violation behavior. Participants then reported how they felt about the norm violation by ticking the box of any emotions that applied from a list of nine emotions. Following this, participants rated the appropriateness of different sanctions (confrontation, ostracism, and gossip) directed at the violator. Five norm violation scenarios were presented with the same procedure.

Following the preregistered plan (link provided in “Methods”), we first examined the correlation between the emotions of disgust and anger. The correlation was not very high (averaged across scenarios: Pearson *r* = 0.22, polychoric *r* = 0.39). Thus, these emotions are distinct in the data and may hence have distinct effects on judgments of sanctions.

### Preregistered analysis related to Hypothesis 1

**Figure 1:**
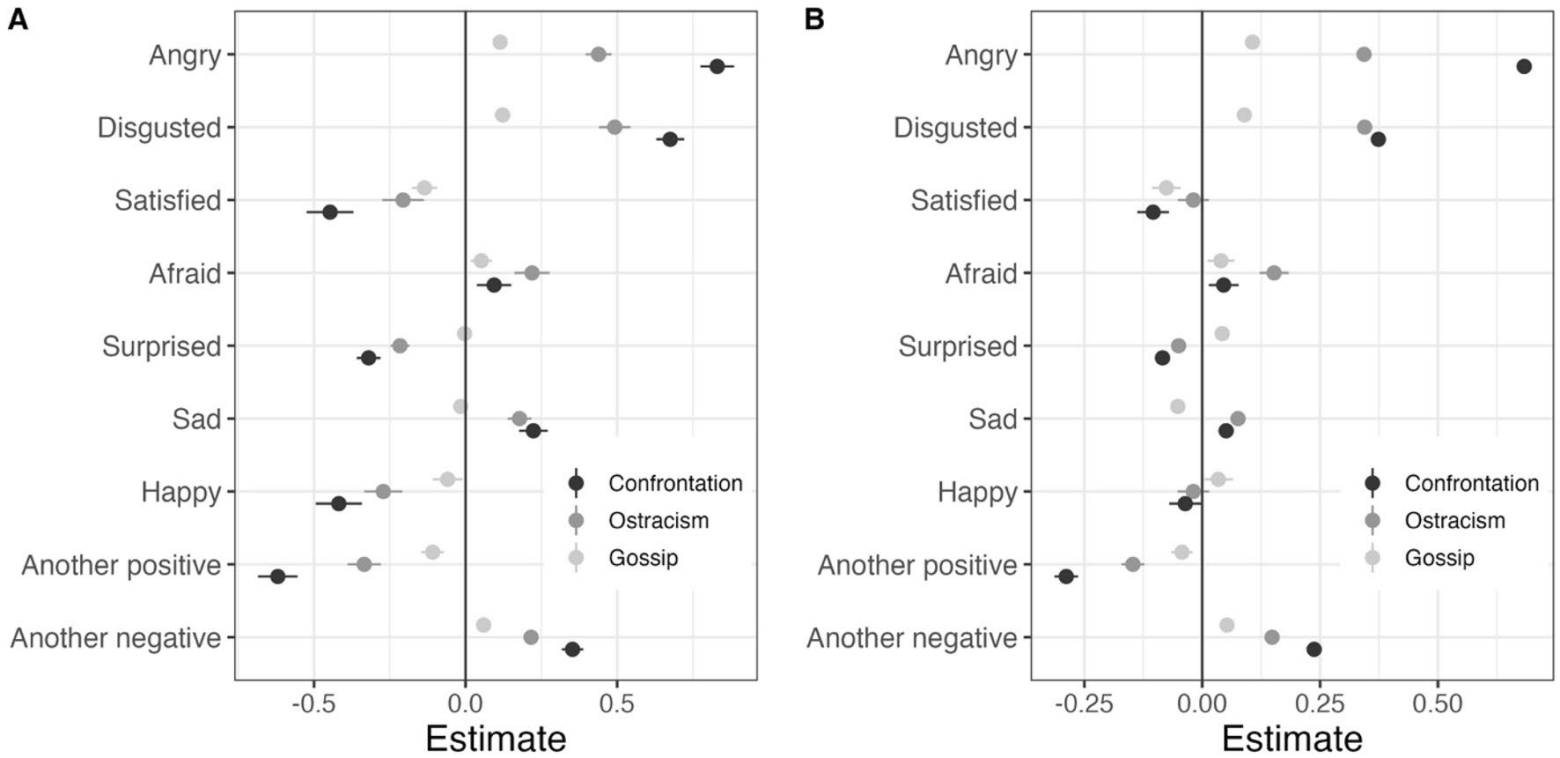
Standardized coefficients of different emotions as predictors of the perceived appropriateness of informal sanctions. A coefficient of 0.5 means that participants who reported the emotion for the scenario tended to perceive the sanction for the scenario as 0.5 standard deviations more appropriate than participants who did not report the emotion. (**A**) Effects estimated separately for each emotion (Model 1). (**B**) Independent effects of each emotion when all emotions were simultaneously included as predictors (unlike Model 1, this model does not include random slopes because that model becomes too complex to estimate).

Hypothesis 1 concerns the global effects of anger and disgust on judgments of the appropriateness of sanctions. Following the preregistered plan, we examined Hypothesis 1 using a given emotion as a predictor and with age and gender as individual-level controls in a mixed-effect regression model with three levels: individual appropriateness ratings of a given type of sanction in five scenarios, nested in 17,774 individuals, nested in 56 countries. For the sake of transparency, here we display all measured emotions in Fig. 1, although the preregistered analyses only concern the emotions of anger and disgust. For each emotion, Fig. 1A shows the estimate of its fixed effect with a 95% confidence interval. As different emotions are correlated with each other, we additionally ran an analysis to examine their independent effects by including all emotions as multiple predictors in the same model (see Fig. 1B). In both analyses we find that anger and disgust are related to more positive judgments of the appropriateness of informal sanctions. Thus, Hypothesis 1 was supported. Figure 1 also displays the estimated effects of emotions other than anger and disgust, allowing an overall comparison of how emotions are linked to judgments of social sanctions. Note that the effects of other negative emotions (feeling afraid, sad, or “another negative emotion”) were substantially smaller than those of anger and disgust, in support of the assumption that anger and disgust play key roles in judgments of sanctions. It is also noteworthy that positive emotions about a norm violation are associated with less positive ratings of sanctions.

The results for anger and disgust in Fig. 1 speak to the debate about whether these emotions motivate different kinds of sanctions. On the one hand, both emotions have positive effects on ratings of all three sanctions. On the other hand, there are dramatic differences in the size of the effects. Ratings of confrontation are strongly predicted by anger and moderately strongly predicted by disgust. Ratings of ostracism are moderately strongly predicted by disgust as well as by anger. Both emotions have much smaller effects on ratings of gossip.

### Additional non-preregistered analyses related to Hypothesis 1

**Figure 2:**
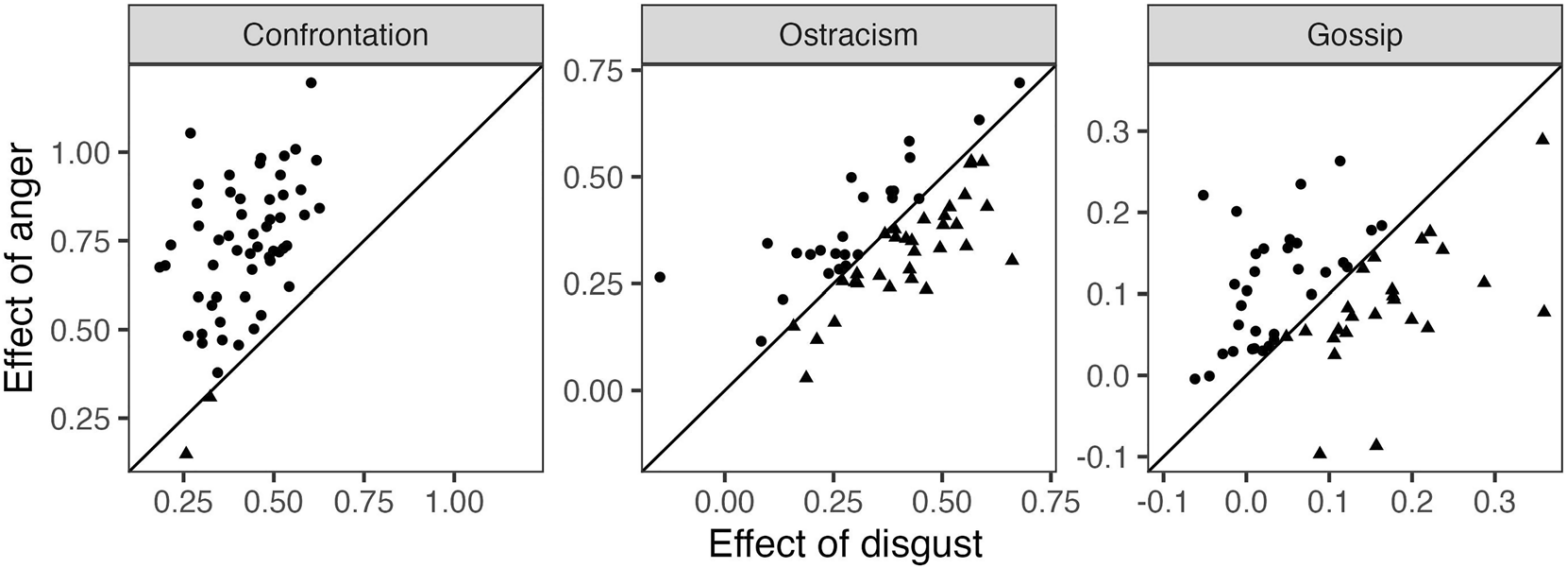
Scatterplots of the effects of anger (*y*-axis) and disgust (*x*-axis) on ratings of sanctions reflected in standardized coefficients. Effects were estimated separately in each of the 56 countries for each type of sanction (confrontation, ostracism, and gossip) in models that included random intercepts for individuals and adjusted for gender and age. Dots (vs. triangles) that is above (vs. below) the diagonal *y* = *x* represent countries where the effect of anger was larger (vs. smaller) than the effect of disgust.

To further examine the difference between anger and disgust, we performed additional data contingent analyses in which we simultaneously included anger and disgust as predictors of ratings of a given type of sanction separately in each country (reducing the number of levels to two: five scenarios nested in individuals). Results are summarized in scatterplots in Fig. 2. Here, dots represent countries where the effect of anger (*y*-axis) was larger than the effect of disgust (*x*-axis), and triangles represent countries where the opposite held. Each of the 56 countries thus appear as either a dot or triangle. Note that the ratings of confrontation are, almost universally, more affected by anger than by disgust, with only two triangles in the first panel. By contrast, ratings of ostracism are slightly more often more affected by disgust than by anger, with 32 triangles and 24 circles. These findings lend only weak support to anger and disgust being predictive of different behavioral preferences. Regarding Hypothesis 1 more generally, the directions of the country-level effects show further support for universality. That is, for ratings of confrontation, the estimated effects of anger and disgust were positive in every country. This also held for ratings of ostracism, with a single exception for the effect of disgust. Thus, the phenomenon that confrontation and ostracism are judged as more appropriate when individuals feel angry or disgusted with the norm violation seems to hold essentially everywhere.

### Preregistered analysis related to Hypothesis 2

**Figure 3:**
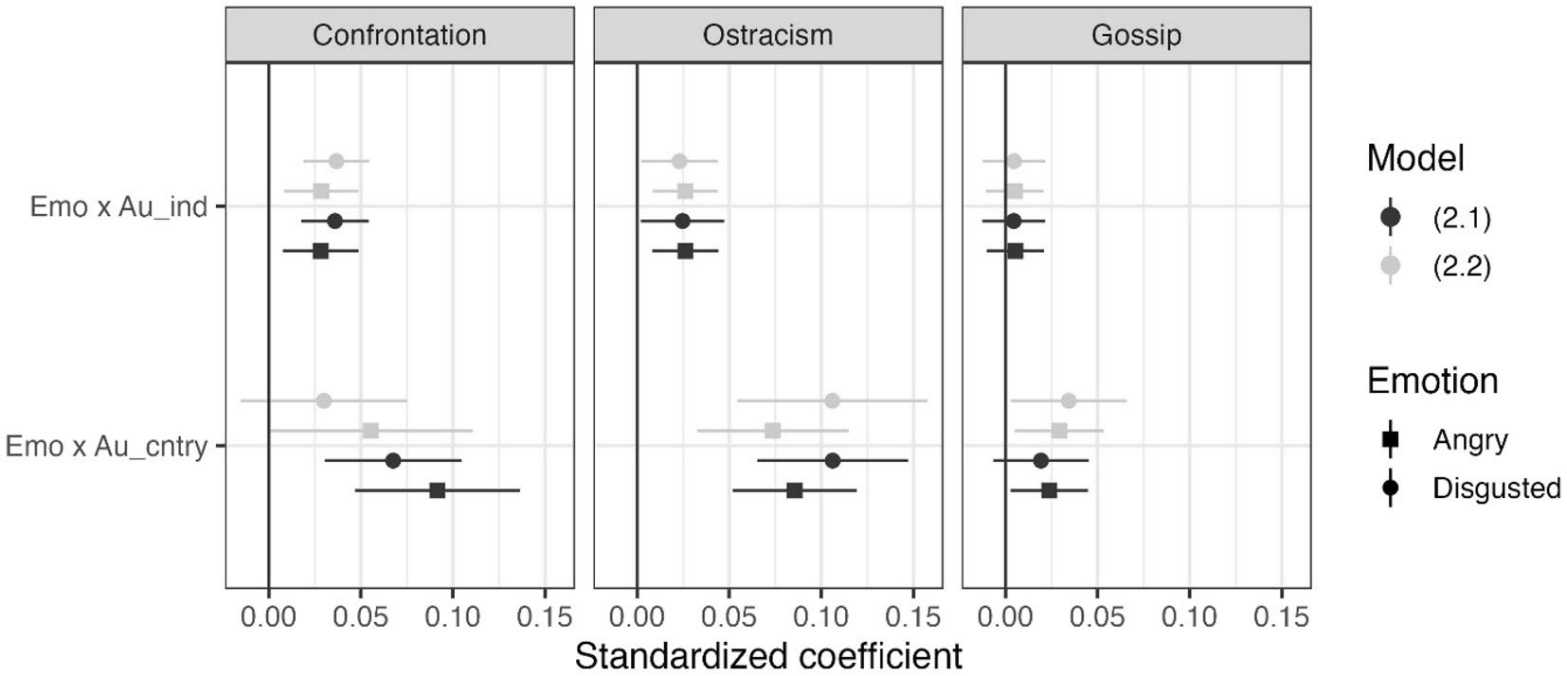
Estimates (standardized coefficients) of how the association between emotion and the appropriateness of different sanctions is moderated by autonomy at individual level (Emo × Au_ind) and at the country level (Emo × Au_cntry). Estimates were obtained from three-level models (Model 2 in the “Methods” section) with different sets of controls. Model 2.1 includes only individual-level controls, whereas Model 2.2 (preregistered) additionally includes country-level measures of human development (income and education) and their interactions with emotion.

Figure 2 illustrates that the effect of emotions on judgments of sanctions varies across countries. Hypothesis 2 posits that some of this cross-country variation can be explained by cultural differences in individual autonomy. To examine this hypothesis, we preregistered analyses including individual-level and country-level measures of autonomy values and their interactions with the given emotion as predictors, with measures of economic and educational development as country-level controls. Figure 3 reports estimates of how the effect of an emotion (Emo) is moderated by country-level autonomy (Au_cntry) and individual-level autonomy (Au_ind). In support of Hypothesis 2, these interaction effects are consistently positive (although in a few cases the confidence interval includes zero). In other words, higher valuations of individual autonomy are associated with emotions playing a larger role in guiding people’s judgment about sanctions.

### Additional non-preregistered analyses related to Fig. 3

**Figure 4:**
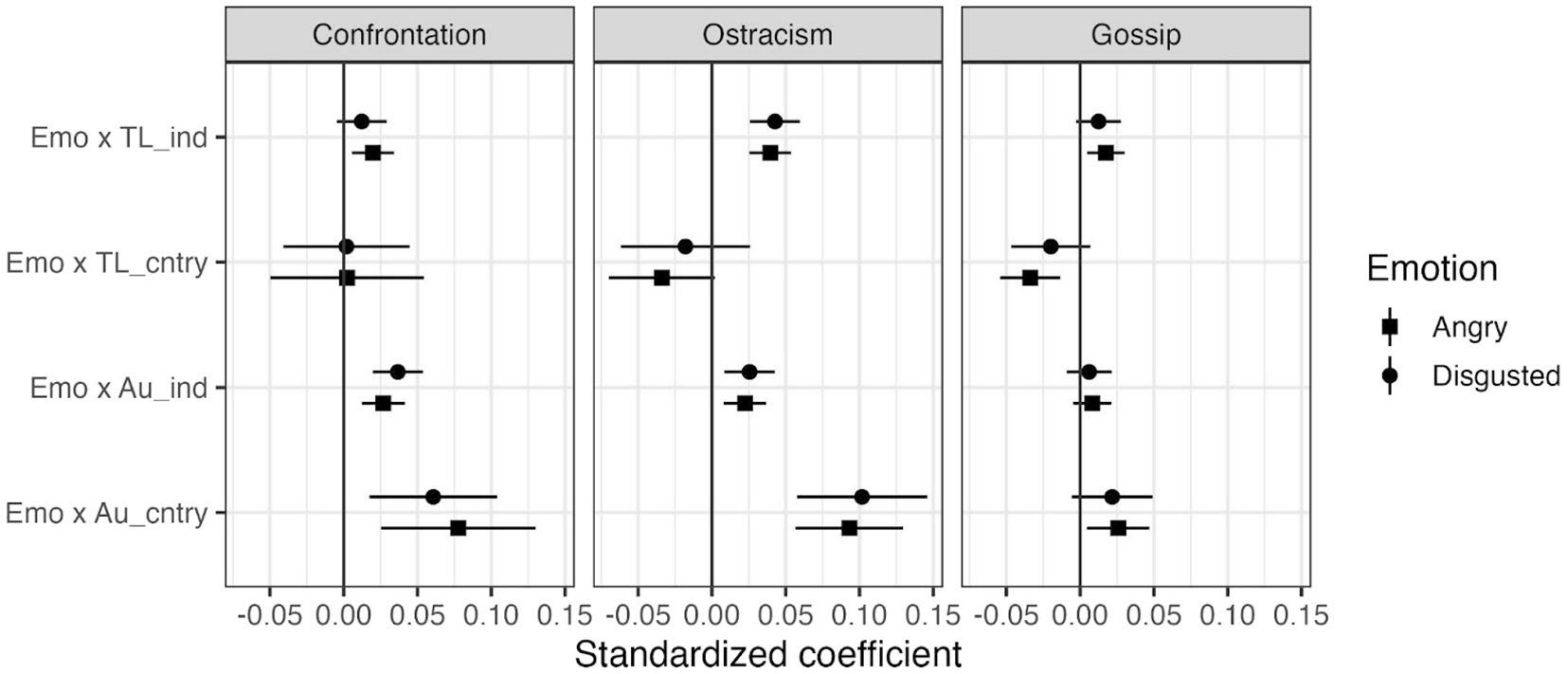
Estimates (standardized coefficients) of how the association between emotion and the appropriateness of different sanctions is moderated by autonomy and tightness/looseness at individual level (Emo × Au_ind and Emo × TL_ind) and at the country level (Emo × Au_cntry and Emo × Au_cntry). Tightness/looseness is shown as the degree of Looseness. Estimates were obtained from three-level models with individual level controls (unlike Model 2, this model does not include random slopes for Au_ind and Emo × Au_ind).

The construct of tightness/looseness^43^ refers to cultures having stronger or looser norms and norm enforcement, and can be seen as theoretically related to autonomy as measured here. Cultural looseness is partly correlated to autonomy in our data (Pearson r = 0.47). In order to investigate whether the autonomy index used here is separable from other related constructs, such as Gelfand’s et al.^43^ tightness/looseness, we also include an additional statistical model. Figure 4 illustrates the same model as Fig. 3, but with the addition of Gelfand’s tightness/looseness within the model. Figure 4 thus report estimates of how the effect of emotion (Emo) on judgments of sanctions is moderated by country-level autonomy (Au_cntry) and individual-level autonomy (Au_ind). Even here, as Gelfand’s tightness/looseness is included into the model, Autonomy appears to still moderate the effect of emotion on judgments of confrontation and ostracism. Gelfand’s cultural looseness also show a similar pattern as autonomy on the individual level, but not the country level. That is, individuals who rate their own culture as being less strict in norms and enforcement are somewhat more likely to have their emotions play a larger role in guiding their judgments of sanctions. Autonomy values and cultural looseness thus appear to have distinct effects, in the same direction, at the individual level.

### Additional non-preregistered analyses related to Hypothesis 2

**Figure 5:**
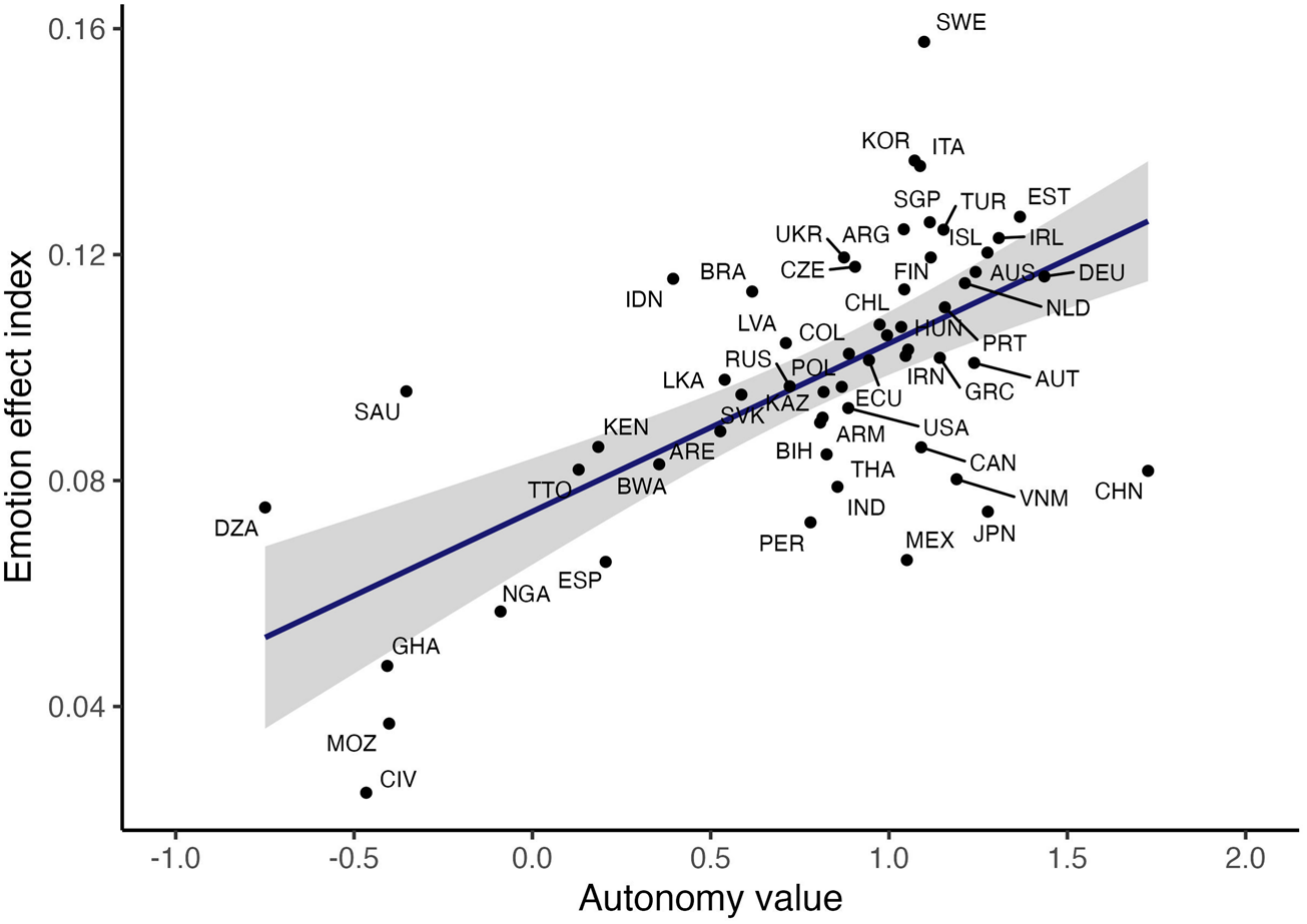
Scatterplot of the general emotion effect against country-level autonomy values in 56 countries, including regression line with 95% confidence interval for the slope. Dots are labeled by ISO country codes.

Recall the theoretical reasoning behind Hypothesis 2: higher autonomy leads to individuals’ judgments of sanctions being less influenced by authorities, and hence individuals’ own emotions will play a more important role in their judgments. It is worth noting that this line of argument is not specific to the emotions of anger and disgust, nor is it specific to judgments of sanctions. Indeed, this line of argument suggests a more general phenomenon: all judgments regarding appropriate behavior should be more strongly predicted by emotions in high-autonomy cultures than in low-autonomy cultures. To test this more general prediction, we analyze how well all emotions in the study (nine emotions in total) together predict each of the 25 judgments that participants made (each of five scenarios rated five times: the norm violation itself, confrontation, ostracism, gossip, and the additional option of non-action in response to the norm violation), separately in each country. For each country, this yields a set of 25 values of R^2^, that is, proportions of variance in judgments explained by emotions. We average these to a single country index of the general emotion effect on appropriateness judgments (α = 0.81). Figure 5 plots the emotion effect index against country-level autonomy values. The strong positive correlation, *r* = 0.63, indicates that people in countries with higher autonomy values are more affected by emotions when judging the appropriateness of norm violations and responses to norm violations. As a robustness check, we verified that the emotion effect index also correlates with each separate component of the autonomy measure: *r* = 0.67 for low valuation of obedience, *r* = 0.57 for low valuation of religious faith, *r* = 0.46 for high valuation of independence, and *r* = 0.23 for high valuation of determination. Overall, our ranking of high and low autonomy values in countries is consistent with similar datasets (see details in the “Methods” section).

## Discussion

In a large and culturally diverse sample, we examined how direct emotions about a norm violation influence judgments of the appropriateness of sanctions. Compared to other emotions, feelings of anger and disgust were much stronger predictors of appropriateness judgments of confrontation and ostracism. That anger and disgust are key emotions in judging the use and appropriateness of sanctions is consistent with prior literature^23,25,39^. Our study contributes to this literature by demonstrating that the phenomenon is not limited to WEIRD^26^ countries, as it appears to exist across a wide range of countries. Specifically, our study shows this phenomenon occurs in our sample of relatively mild norm violations, thus demonstrating a similar pattern to more severe and moralized norm violations from previous studies. It is likely that people’s use of social sanctions is sensitive to how appropriate such sanctions are judged by others. Our study therefore indicates that use of sanctions could depend not only on the extent to which the individual who is in position to deliver the sanction feels anger and disgust, but also on the anger and disgust that bystanders feel. Thus, emotions have both direct and indirect effects on sanctioning.

Our study also speaks to the question of whether specific sanctions are motivated by specific emotions^44^. We found this to hold only in a weak sense, because both anger and disgust had independent positive effects on ratings of all sanctions that we studied. Nonetheless, ratings of confrontation were always more strongly predicted by anger, which is consistent with prior studies of sanctions^23^. Ratings of ostracism and gossip were, however, not more strongly predicted by disgust in our study. Naturally, for there to be a relation between sanction and an emotion, the prior norm violation must elicit the emotion first, and some violations may elicit stronger anger than disgust, or vice versa^45,46^. That the relation between emotions and ratings of sanctions appear weaker for gossip could potentially be due to how gossip does not fit as neatly into an approach and avoidance framework. For example, by being an active action targeting the norm violator’s reputation, gossip has elements of approaching, yet it involves avoiding direct contact with the norm violator, giving it elements of avoidance as well.

Based on the idea that there are both external and internal sources of guidance for judgments^41^, together with the notion that tradition and religion provide such external sources, we made the novel hypothesis that the effect of emotions should be stronger in countries that promote individual autonomy. Consistent with this hypothesis, autonomy values were associated with a greater reliance on emotions in appropriateness judgments of sanctions. This finding is related to, but distinct from, prior findings of greater endorsement of emotional display in more individualistic cultures^47^. Similarly, this finding is distinct but partly related to recent studies on how cultural individualism may lead to greater homogeneity in valued and experienced emotions^48^. Autonomy, as a general term, relates to several constructs measured in studying cultural differences. Here we included Gelfand et al.’s tightness/looseness^43^ construct and found that while it was partly correlated to our autonomy index, our main findings remained when both were included in the same model. In terms of differences, the autonomy index we used, based on the World Values Survey, is about values individuals find important when raising children, while Gelfand's construct relates to how values are enforced and practiced within a society. Other constructs related to autonomy not measured here include CADS^49^, based on Shweder’s moral codes. Future research will be needed to further tease apart different aspects of autonomy values, and how these values could relate to the role emotions play in judging sanctions for norm violations.

Our finding was obtained not only at the country level but also at the individual level, providing additional evidence that the relation is not spurious. It supports the theory that individuals who exercise autonomy by making judgments about appropriate behavior on their own rather than sourcing them from authorities and traditions will rely more on their own emotions. Note that this mechanism is not limited to the emotions of anger and disgust or to judgments of informal sanctions. We found the same moderating effect of autonomy for all emotions in our study and for judgments of norm violations as well as sanctions. Thus, the interaction between autonomy and emotions may have a wide scope. Indeed, it is not clear whether traditional or religious authorities would provide clear guidance on the specific scenarios used in the present research. Our interpretation is that low autonomy makes people more likely to rely on such authorities while high autonomy promotes a habitual reliance on emotional intuitions. Other research indicate that autonomy values exhibit a rising trend across time^42,50–52^. Our findings suggest that this trend may be associated with increasingly emotion-led judgments of appropriate behavior. Whether such a trend is positive or not for a society is open for debate. Another question, beyond the scope of the present study, is whether people in high-autonomy cultures are also more likely to consider it right to base sanctions on emotions.

Some strengths and limitations of the present research should be noted. First, in the present study, we investigated only individual-level state-based emotions and thus cannot generalize to collective emotions or trait-based emotional differences. We thus make no claims about differences in emotional capacities, but rather about the relation between state-based emotions and judgments about appropriate behavior. Emotion is a broad concept, and future studies are needed to fill out the full picture. Second, there are cultural differences in how emotions are labelled across language, and what kind of behaviors and associations that are connected to emotional words^29^. A limitation of this kind of study is that we cannot capture all the nuances of how emotions are constructed within specific cultural settings. The stability of the results across cultures in this study tells us about similarities, but further studies are needed to highlight underlying differences. For example, cultural differences in how specific sanctions are seen as more or less effective within a culture can also play a part in moderating the findings here. Third, the large number of countries and participants in the present study allow us to make relatively precise estimates of the effects of individual-level state-based emotions on appropriateness judgments and how these effects vary across countries. However, our samples are not demographically representative of each country as data were collected in cities and mainly among students. Finally, while a strength of the study is that a variety of norm violation scenarios were included, these scenarios were hypothetical. Conclusions from this research therefore rest on the assumption that the process by which people make judgments in general is the same as the process of when they rate hypothetical scenarios. The scenarios used in this study depicted relatively mild violations of conventional norms, and thus extends similar findings from more severe moral violations to these milder scenarios. Under these assumptions, a major contribution of the present research is the finding that the effect of emotions on judgments of appropriate sanctions is universal, but is moderated by cultural differences in individual autonomy.

## Methods

The data on emotions and appropriateness ratings analyzed in this study were collected as part of the International Study of Metanorms (ISMN). While appropriateness ratings were analyzed in the main paper on this project^8^, the data on emotions were previously unpublished. After data collection, the only researcher with access to the data was K.E. (last author on the present paper). He invited P.A.A., I.V., D.V., and G.T. (the first four authors of the present paper), who had no access to the data, to independently develop hypotheses and analysis methods and to preregister them prior to data analyses. The preregistration plan can be found at https://doi.org/10.17605/OSF.IO/48CYN.

The research was conducted in accordance with relevant guidelines and regulations, including the Declaration of Helsinki. All participating countries approved the study protocol, which detailed the methods, procedure and variables collected. Approval of the study protocol was obtained from ethics committees and institutional review boards where required, including Queen’s University (Canada), York University (Canada), Bogotá (Colombia), Institute of Psychology at the Czech Academy of Sciences (Czech Republic), Universidad San Francisco de Quito (Ecuador), United Psychological Research Committee (Hungary), Monk Prayogshala (India), the Trinity College Dublin School of Social Sciences and Philosophy (Ireland), Kwansei Gakuin University (Japan), Aoyama Gakuin University (Japan), United States International University—Africa (Kenya), Sunway University (Malaysia), University of Amsterdam (Netherlands), Komisja ds. Etyki Badań Naukowych Wydziału Psychologii Uniwersytetu SWPS (Poland), Instituto de Ciências Sociais (Portugal), Doha Institute for Graduate Studies (Qatar), Singapore Management University (Singapore), Sungkyunkwan University (South Korea), Universidad de Navarra (Spain), Post Graduate Institute of Medicine (Sri Lanka), Chulalongkorn University (Thailand), American University of Sharjah (United Arab Emirates), University of Kent (United Kingdom), Brunel College of Health and Life Sciences (United Kingdom), University of South Carolina (United States), and New York University (United States).

### Participants and procedure

The International Study of Metanorms (ISMN) was the collaboration that involved the data collection for this study, as well as the previously published article by Eriksson et al.^8^. The ISMN data collection was based on a survey that was translated to 30 different languages, using independent translation and back-translation. Data was collected in 57 countries from April 2019 to January 2020. One country later chose to opt out of this project and thus we only used data from 56 countries. Recruitment methods varied between locations, including methods such as invitations by email, in class, on social media, using public notes, using survey organizations, and face to face on campus recruitment. The goal of the data collection was for each country to collect data from approximately 200 students or more, which was achieved in almost all countries, with 31 countries also collecting data from non-student samples. Data was primarily collected at major cities, as this is where most universities and colleges are located.

Participants took the survey anonymously using the online survey provider Qualtrics, with exceptions for participants in Ghana and non-students in Estonia, who completed the survey on paper instead. The survey started with a standard informed consent form, and all participants gave their informed consent. The survey took approximately 30 min, and the stated purpose was an international study of social norms.

The total number of participants in the ISMN was over 22,000 participants. After excluding missing values for the key variables, age, and those with unknown gender, we analyzed data from the 17,774 participants. The gender distribution was somewhat uneven (66% women, 34% men). Most participants were students (80%) so the sample was overall quite young (mean age of 24.9 years with a standard deviation of 8.9). Sample sizes per country ranged from 45 to 995 with a median of 292. Supplementary Table S1 presents the exact set of countries and sample characteristics of each country.

### Measurements and materials

Below, we present only the variables that are used in the current study. The full survey can be accessed at OSF (https://osf.io/pm5kc/), together with the data we analyze here.

#### Scenarios

Five scenarios presented different norm violations. The first scenario presented a violation of a cooperation norm in the form of an animation of an agent depleting a common resource, which has been used in prior research^7^. Subsequent scenarios in written form presented four out-of-place everyday behaviors that have previously been tested and widely seen as inappropriate across cultures^43^: a person sleeping in a restaurant, singing in a library, listening to music on headphones at a funeral, and reading a newspaper at the movies.

#### Outcome variables

For each of the five norm violation scenarios, participants rated the norm violation as well as four possible responses to it: doing nothing, confrontation, ostracism, and gossip. Participants indicated how appropriate the response was on a 6-point continuous scale from “extremely inappropriate” to “extremely appropriate”. Our main analysis focused on the three latter responses, which represented various forms of informal sanctions.

#### Predictor variables

Emotional responses were measured by having participants check a box of all emotions that they felt in response to the norm violation. These were: “happy”, “sad”, “surprised”, “afraid”, “disgusted”, “angry”, “satisfied”, “another positive emotion” and “another negative emotion”. In our main analyses, we only used “disgusted” and “angry”. The check box method suggested that these were binary variables, and no text input was allowed for the option of another emotion.

Autonomy values was an index created from a set of ISMN items that were adapted from the World Values Survey. Participants were presented with a list of 10 important child qualities, out of which they choose up to 5 out of 10 items that they think are especially important. Items relating to higher autonomy is “Independence” and “Determination” (each adding + 1 to score) while “Religious faith” and “Obedience” relate to lower autonomy (each subtracting − 1 from score), creating an individual score ranging from 2 to − 2. Other items on the list were not counted towards the score. The mean score among participants in a country is used as a country-level measure of autonomy values. Supplementary Table S2 presents the country-level measures of autonomy values and its four components. To validate our ranking of high and low autonomy values in countries, other datasets which include the same countries can be used as reference points. For example, data collected in 2017–2022 in the large-scale World Values Survey^53^ show high similarity in the relevant countries and the regions they are from. In the World Values Survey dataset the highest scoring countries in autonomy values also included in our study are, in descending order, Japan, Iceland, South Korea, Sweden, China, Latvia, Austria, Germany, Finland, and Canada. On the other end, the countries lowest in autonomy values in descending order are Nigeria, Ecuador, Kenya, Mexico, Indonesia, Peru, Brazil, Turkey, Armenia, and Argentina. This appears to be consistent with our scoring (as seen in Fig. 4 and Table S2 in the Supplementary Material).

Tightness/looseness is measured with Gelfand’s 6-item tightness scale^43^, with items like “There are many social norms that people are supposed to abide by in this country.” In the original study, responses were standardized by subtracting participants’ mean response to all items in the survey, which was strongly dominated by items on the appropriateness of various behaviors in various contexts. Following this procedure, we adjusted the responses to the tightness items in our survey by subtracting participants’ mean response to all appropriateness items.

#### Control variables

Individual-level controls were gender and age. Participants were asked for their gender (male, female, other/do not want to say). In the analysis we included only participants who answered male or female. Age was measured as a self-reported number. Country-level controls were the income and education components of the Human Development Index, provided by the United Nations.

### Data analysis

Model 1 is written as follows:

$${\text{App}}_{ijk} = \left( {\beta_{{1}} + u_{{{1}jk}} + v_{{{1}k}} } \right) + \left( {\beta_{{2}} + u_{{{2}jk}} + v_{{{2}k}} } \right){\text{Emo}}_{ijk} + \beta X_{jk} + {\text{error}}_{ijk} .$$

here, App_*ijk*_ and Emo_*ijk*_ denote the ratings of appropriateness and the emotion dummy, respectively, of behavior *i*, as rated by individual *j* in country *k*, standardized to have global mean zero and unit standard deviation. Note that β_2_ represents the emotion-appropriateness association. Random effects at the individual level (*u*_1*jk*_, *u*_2*jk*_) and the country level (*v*_1*k*_, *v*_2*k*_) follow a multivariate normal distribution with a mean of zero. Finally, *X*_*jk*_ is an individual-level control variable.

Model 2 is written as follows:

$$\begin{aligned} {\text{App}}_{ijk} & = \left( {\beta_{{1}} + u_{{{1}jk}} + v_{{{1}k}} } \right) + \left( {\beta_{{2}} + u_{{{2}jk}} + v_{{{2}k}} } \right){\text{Emo}}_{ijk} + \left( {\beta_{{3}} + v_{{{3}k}} } \right){\text{Au}}\_{\text{ind}}_{jk} + \left( {\beta_{{4}} + v_{{{4}k}} } \right){\text{Emo}}_{ijk} \\ & \quad \times {\text{Au}}\_{\text{ind}}_{jk} + \beta_{{5}} {\text{Au}}\_{\text{cnt}}_{k} + \beta_{{6}} {\text{Emo}}_{ijk} \times {\text{Au}}\_{\text{cnt}}_{k} + \beta X_{ijk} + {\text{error}}_{ijk} . \\ \end{aligned}$$

here, App_*ijk*_ and Emo_*ijk*_ denote the ratings of appropriateness and emotion, respectively, of behavior *i*, as rated by individual *j* in country *k*, standardized to have a global mean of zero and unit standard deviation. *Au_ind*_*jk*_ represents the same individual’s autonomy score, standardized to have a global mean of zero and unit standard deviation. *Au_cnt*_*jk*_ is the country-level mean of *Au_ind*_*jk*_ (standardized again). In this paper, we focus on three coefficients: β_2_ measures the emotion-appropriateness association, β_4_ measures how the emotion-appropriateness association is moderated by individual autonomy, and β_6_ measures how the emotion-appropriateness association is moderated by country-level autonomy. Random effects at the individual level (*u*_1*jk*_, *u*_2*jk*_) and the country level (*v*_1*k*_, *v*_2*k*_, *v*_3*k*_, *v*_4*k*_) follow a multivariate normal distribution with a mean of zero. Finally, *X*_*ijk*_ represents terms representing individual-level controls, country-level controls, and cross-level interactions between controls and *Emo*_*ijk*_.

### Supplementary Information

Supplementary Information.

## Data Availability

The datasets analyzed during the current study are available in the Center for Open Science repository, https://osf.io/djnfg/.
