# Supplementary material for: Anger and disgust shape judgments of social sanctions across cultures, especially in high individual autonomy societies
Source: Sci Rep. 2024 Mar 7;14:5591. doi: 10.1038/s41598-024-55815-x (PMC10920647; doi:10.1038/s41598-024-55815-x)

Anger and disgust shape judgments of social sanctions across cultures, especially in high individual autonomy societies

Authors and affiliations

Per A. Andersson[*1,2], Irina Vartanova[3], Daniel Västfjäll[1], Gustav Tinghög[2,4], Pontus Strimling[3], Junhui Wu[5,80], Isabela Hazin[3,81], Charity S. Akotia[6], Alisher Aldashev[7], Giulia Andrighetto [3,8,9], Adote Anum[6], Gizem Arikan[10], Fatemeh Bagherian[11], Davide Barrera[12], Dana Basnight-Brown[13], Birzhan Batkeyev[14], Elizaveta Berezina[15], Marie Björnstjerna[3], Paweł Boski[16], Inna Bovina[17], Bui Thi Thu Huyen[18], Đorđe Čekrlija[19], Hoon-Seok Choi[20], Carlos C. Contreras-Ibáñez[21], Rui Costa-Lopes[22], Mícheál de Barra[23], Piyanjali de Zoysa[24], Angela R. Dorrough[25], Nikolay Dvoryanchikov[17], Jan B. Engelmann[26], Hyun Euh[27], Xia Fang[28], Susann Fiedler[29], Olivia A. Foster-Gimbel[30], Márta Fülöp[31,32], Ragna B. Gardarsdottir[33], C. M. Hew D. Gill[15], Andreas Glöckner[34,35], Sylvie Graf[36], Ani Grigoryan[37], Vladimir Gritskov[38], Katarzyna Growiec[16], Peter Halama[39], Andree Hartanto[40], Tim Hopthrow[41], Martina Hřebíčková[36], Dzintra Iliško[42], Hirotaka Imada[43], Hansika Kapoor[44], Kerry Kawakami[45], Narine Khachatryan[37], Natalia Kharchenko[46], Toko Kiyonari[47], Michal Kohút[48], Lisa M. Leslie[30], Yang Li[49], Norman P. Li[40], Zhuo Li[50], Kadi Liik[51], Angela T. Maitner[52], Bernardo Manhique[53], Harry Manley[54,79], Imed Medhioub[55], Sari Mentser[56], Pegah Nejat[11], Orlando Nipassa[53], Ravit Nussinson[56,57], Nneoma G. Onyedire[58], Ike E. Onyishi[58], Penny Panagiotopoulou[59], Lorena R. Perez-Floriano[60], Minna Persson[3], Anna-Maija Pirttilä-Backman[61], Marianna Pogosyan[62], Jana Raver[63], Ricardo Borges Rodrigues[64], Sara Romanò[65], Pedro P. Romero[66], Inari Sakki[67], Alvaro San Martin[68], Sara Sherbaji[52,69], Hiroshi Shimizu[70], Brent Simpson[71], Erna Szabo[72], Kosuke Takemura[73], Maria Luisa Mendes Teixeira[74], Napoj Thanomkul[54], Habib Tiliouine[75], Giovanni A. Travaglino[76], Yannis Tsirbas[77], Sita Widodo[78], Rizqy Zein[78], Lina Zirganou-Kazolea[77], Kimmo Eriksson[3,8]

Supplementary Material

Contents

Supplementary Material 1

Supplementary Table S1. Sample characteristics in each country. 3

Supplementary Table S2. Autonomy means (sorted from highest to lowest) and separate index components scores across 56 countries. 5

Supplementary Table S3. Means and standard deviations for selecting the emotions angry or disgusted in response to scenarios. 7

Supplementary Table S4. Proportion of participants who selected a given emotion. 7

Supplementary Figure SF1. Average number of emotions selected. 8

Supplementary Table S5. Average reported anger and disgust across countries, mean and standard deviation (in parentheses). 9

Supplementary Table S6. Average number of emotions selected per country. 11

Supplementary Table S7. Correlations between autonomy and other variables 18

Supplementary Figure SF2. Model depicted in the main manuscript as Figure 3, here with Tightness replacing Autonomy. 18

Supplementary Figure SF3. Model depicted in the main manuscript as Figure 1, here including the ratings of appropriateness in the same model. 19

Supplementary Table S1. Sample characteristics in each country.

Country	*N*	% female	M_{age} (*SD*)	% students
Algeria	70	79	23.2 (5.3)	100
Argentina	443	69	25.9 (9.2)	47
Armenia	340	67	24.0 (6.3)	67
Australia	248	76	19.7 (3.8)	100
Austria	339	67	22.0 (3.5)	100
Bosnia and Herzegovina	236	50	21.2 (2.1)	100
Botswana	45	82	20.9 (1.5)	100
Brazil	280	55	31.4 (13.0)	69
Canada	425	67	20.3 (3.8)	100
Chile	127	43	20.9 (1.5)	100
China	995	70	22.5 (6.7)	80
Colombia	341	60	25.6 (9.4)	67
Czech Republic	375	77	28.3 (9.2)	69
Ecuador	283	62	22.7 (5.2)	84
Estonia	341	79	29.6 (9.2)	65
Finland	232	81	30.6 (11.1)	89
Germany	654	69	28.9 (10.4)	30
Ghana	278	57	23.0 (7.0)	78
Greece	500	69	27.3 (10.7)	73
Hungary	449	80	24.5 (6.6)	79
Iceland	464	77	30.7 (11.8)	78
India	259	88	19.7 (4.8)	96
Indonesia	304	82	22.2 (6.4)	69
Iran	221	72	21.4 (3.7)	100
Ireland	237	58	22.1 (8.5)	95
Israel	334	60	27.8 (5.0)	68
Italy	384	59	22.9 (4.4)	100
Ivory Coast	186	43	26.3 (5.8)	100
Japan	557	53	19.9 (2.9)	100
Kazakhstan	182	61	19.7 (5.4)	100
Kenya	189	49	22.0 (2.7)	100
Latvia	327	72	29.9 (9.8)	89
Malaysia	339	53	25.1 (8.3)	69
Mexico	140	69	25.2 (7.6)	100
Mozambique	200	36	23.2 (4.8)	100
Netherlands	269	55	21.7 (4.4)	100
Nigeria	300	57	27.9 (8.4)	61
Peru	257	68	33.7 (12.9)	41
Poland	543	71	35.2 (13.5)	44
Portugal	140	89	24.3 (7.1)	100
Russia	381	77	22.7 (6.3)	100
Saudi Arabia	304	25	24.6 (8.7)	71
Singapore	201	69	22.0 (1.6)	100
Slovakia	353	56	30.1 (10.0)	66
South Korea	362	55	27.3 (10.2)	65
Spain	252	43	30.5 (12.6)	52
Sri Lanka	188	62	23.2 (1.9)	100
Sweden	203	54	27.2 (9.3)	100

Thailand	202	70	19.8 (2.7)	100
Trinidad and Tobago	190	81	26.3 (8.6)	64
Turkey	229	83	21.6 (1.6)	100
UAE	301	64	20.0 (1.3)	100
Ukraine	246	64	29.3 (11.7)	59
United Kingdom	424	84	24.4 (10.3)	71
United States	657	77	20.2 (3.0)	100
Vietnam	448	77	18.7 (1.2)	100

Supplementary Table S2. Autonomy means (sorted from highest to lowest) and separate index components scores across 56 countries.

Country	Autonomy values	Independence	Determination	Religious faith	Obedience
China	1.72	0.88	0.88	0.01	0.03
Germany	1.44	0.90	0.68	0.09	0.05
Estonia	1.37	0.86	0.72	0.01	0.20
Ireland	1.31	0.73	0.70	0.07	0.06
Japan	1.28	0.77	0.60	0.02	0.08
Iceland	1.27	0.88	0.52	0.02	0.11
Australia	1.24	0.75	0.72	0.09	0.13
Austria	1.24	0.89	0.63	0.12	0.16
Netherlands	1.21	0.77	0.58	0.06	0.07
Vietnam	1.19	0.70	0.63	0.03	0.11
Portugal	1.16	0.67	0.69	0.06	0.14
Turkey	1.15	0.64	0.58	0.04	0.03
Greece	1.14	0.71	0.73	0.14	0.14
Finland	1.12	0.57	0.63	0.04	0.04
Singapore	1.11	0.68	0.74	0.19	0.11
Sweden	1.10	0.82	0.39	0.02	0.08
Canada	1.09	0.68	0.73	0.16	0.16
Italy	1.09	0.62	0.67	0.04	0.16
South Korea	1.07	0.51	0.66	0.08	0.01
United Kingdom	1.05	0.67	0.68	0.14	0.15
Mexico	1.05	0.66	0.49	0.07	0.04
Iran	1.05	0.67	0.74	0.34	0.02
Argentina	1.04	0.65	0.53	0.05	0.08
Chile	1.04	0.55	0.68	0.05	0.14
Hungary	1.03	0.48	0.77	0.10	0.11
Israel	0.99	0.73	0.60	0.28	0.07
Malaysia	0.97	0.71	0.63	0.18	0.19
Ecuador	0.94	0.56	0.60	0.13	0.10
Czech Republic	0.90	0.48	0.58	0.07	0.09
Colombia	0.89	0.55	0.61	0.16	0.11
United States	0.89	0.59	0.68	0.22	0.17
Ukraine	0.87	0.46	0.69	0.04	0.24
Poland	0.87	0.56	0.55	0.15	0.09
India	0.86	0.66	0.51	0.11	0.20
Thailand	0.83	0.18	0.84	0.03	0.16
Armenia	0.81	0.54	0.54	0.19	0.08
Kazakhstan	0.80	0.41	0.67	0.07	0.21
Bosnia and Herzegovina	0.80	0.50	0.69	0.17	0.22
Peru	0.78	0.57	0.58	0.15	0.22
Russia	0.72	0.39	0.57	0.02	0.22
Latvia	0.71	0.48	0.66	0.17	0.26
Brazil	0.61	0.68	0.53	0.31	0.29
Slovakia	0.58	0.40	0.58	0.16	0.23
Sri Lanka	0.54	0.60	0.48	0.27	0.27
UAE	0.52	0.67	0.52	0.52	0.15

Indonesia	0.39	0.72	0.46	0.64	0.15
Botswana	0.36	0.60	0.58	0.44	0.38
Spain	0.21	0.32	0.56	0.40	0.26
Kenya	0.19	0.51	0.60	0.44	0.48
Trinidad and Tobago	0.12	0.59	0.58	0.52	0.54
Nigeria	-0.09	0.41	0.54	0.46	0.58
Saudi Arabia	-0.36	0.24	0.42	0.68	0.34
Ghana	-0.41	0.33	0.52	0.66	0.60
Mozambique	-0.41	0.32	0.46	0.58	0.62
Ivory Coast	-0.46	0.40	0.48	0.69	0.65
Algeria	-0.76	0.24	0.37	0.81	0.56

Note. The four last columns report the proportion of respondents in each country who selected the corresponding quality as especially important for children to learn at home. The first column, the country score for autonomy values, is given by Autonomy values = (Independence + Determination) - (Religious faith + Obedience).

Supplementary Table S3. Means and standard deviations for selecting the emotions angry or disgusted in response to scenarios.

Scenario	Angry	Disgusted
Listening to music on headphones at a funeral	0.45 (0.12)	0.36 (0.18)
Sleeping in a restaurant	0.09 (0.05)	0.11 (0.08)
Singing in a library	0.56 (0.15)	0.19 (0.15)
Reading a newspaper at the movies	0.12 (0.06)	0.07 (0.07)
Violating a cooperation norm	0.41 (0.11)	0.3 (0.12)

Supplementary Table S4. Proportion of participants who selected a given emotion.

Scenario	Happy	Sad	Surprised	Afraid	Disgusted	Angry	Satisfied	Another Positive	Another Negative
Listening to music on headphones at a funeral	0.01	0.33	0.43	0.03	0.38	0.46	0.02	0.03	0.38
Sleeping in a restaurant	0.03	0.13	0.61	0.04	0.11	0.09	0.03	0.09	0.27
Singing in a library	0.05	0.07	0.45	0.03	0.21	0.55	0.02	0.05	0.38
Reading a newspaper at the movies	0.04	0.05	0.66	0.02	0.07	0.12	0.05	0.09	0.23
Violating a cooperation norm	0.04	0.21	0.46	0.05	0.30	0.41	0.05	0.05	0.40

Supplementary Figure SF1. Average number of emotions selected.

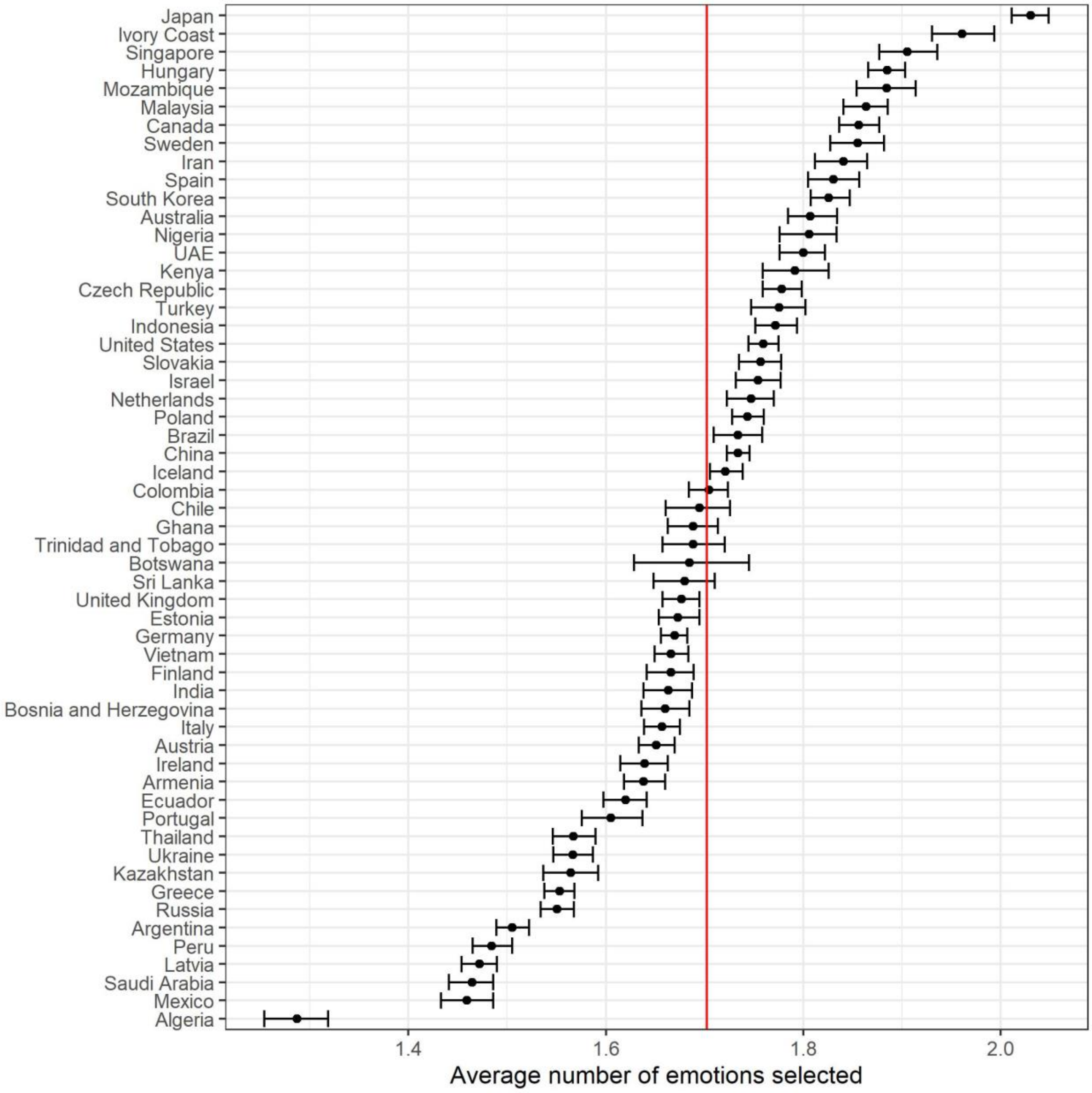

Supplementary Table S5. Average reported anger and disgust across countries, mean and standard deviation (in parentheses).

	Listening to music at a funeral		Sleeping in a restaurant		Singing in a library		Reading a newspaper at the movies		Violating a cooperation norm	
Country	Anger	Disgust	Anger	Disg.	Anger	Disg.	Anger	Disgust	Anger	Disgust
Algeria	0.58 (0.49)	0.26 (0.44)	0.04 (0.20)	0.13 (0.34)	0.43 (0.50)	0.22 (0.41)	0.06 (0.23)	0.16 (0.37)	0.24 (0.43)	0.19 (0.39)
Argentina	0.32 (0.47)	0.13 (0.33)	0.07 (0.26)	0.03 (0.18)	0.64 (0.48)	0.03 (0.17)	0.12 (0.32)	0.01 (0.09)	0.51 (0.50)	0.21 (0.41)
Armenia	0.46 (0.50)	0.23 (0.42)	0.07 (0.26)	0.04 (0.21)	0.51 (0.50)	0.08 (0.28)	0.10 (0.31)	0.06 (0.23)	0.46 (0.50)	0.21 (0.41)
Australia	0.62 (0.49)	0.56 (0.50)	0.08 (0.27)	0.10 (0.30)	0.65 (0.48)	0.23 (0.42)	0.15 (0.36)	0.07 (0.25)	0.43 (0.50)	0.35 (0.48)
Austria	0.65 (0.48)	0.45 (0.50)	0.08 (0.28)	0.17 (0.37)	0.54 (0.50)	0.13 (0.34)	0.05 (0.22)	0.03 (0.17)	0.42 (0.49)	0.28 (0.45)
Bosnia and Herzegovina	0.53 (0.50)	0.53 (0.50)	0.07 (0.26)	0.08 (0.27)	0.51 (0.50)	0.08 (0.27)	0.09 (0.28)	0.02 (0.14)	0.58 (0.49)	0.33 (0.47)
Botswana	0.62 (0.49)	0.44 (0.50)	0.11 (0.31)	0.16 (0.36)	0.67 (0.47)	0.20 (0.40)	0.16 (0.36)	0.09 (0.29)	0.31 (0.46)	0.20 (0.40)
Brazil	0.36 (0.48)	0.13 (0.34)	0.08 (0.27)	0.03 (0.17)	0.70 (0.46)	0.09 (0.28)	0.14 (0.35)	0.04 (0.20)	0.45 (0.50)	0.24 (0.43)
Canada	0.61 (0.49)	0.71 (0.45)	0.11 (0.32)	0.18 (0.38)	0.60 (0.49)	0.21 (0.41)	0.13 (0.34)	0.07 (0.26)	0.41 (0.49)	0.36 (0.48)
Chile	0.42 (0.49)	0.18 (0.39)	0.06 (0.24)	0.06 (0.23)	0.69 (0.46)	0.15 (0.36)	0.08 (0.27)	0.06 (0.23)	0.50 (0.50)	0.25 (0.43)
China	0.54 (0.50)	0.51 (0.50)	0.06 (0.24)	0.10 (0.30)	0.71 (0.46)	0.69 (0.46)	0.06 (0.23)	0.08 (0.26)	0.50 (0.50)	0.52 (0.50)
Colombia	0.43 (0.49)	0.13 (0.34)	0.10 (0.30)	0.08 (0.27)	0.80 (0.40)	0.06 (0.25)	0.15 (0.36)	0.02 (0.13)	0.61 (0.49)	0.22 (0.42)
Czech Republic	0.51 (0.50)	0.60 (0.49)	0.05 (0.21)	0.23 (0.42)	0.49 (0.50)	0.19 (0.40)	0.08 (0.27)	0.07 (0.26)	0.36 (0.48)	0.46 (0.50)
Ecuador	0.47 (0.50)	0.10 (0.29)	0.10 (0.30)	0.05 (0.22)	0.71 (0.45)	0.07 (0.26)	0.17 (0.38)	0.03 (0.18)	0.51 (0.50)	0.23 (0.42)
Estonia	0.18 (0.39)	0.24 (0.43)	0.10 (0.30)	0.06 (0.24)	0.18 (0.38)	0.05 (0.22)	0.16 (0.37)	0.15 (0.36)	0.19 (0.39)	0.19 (0.39)
Finland	0.34 (0.47)	0.41 (0.49)	0.03 (0.18)	0.11 (0.31)	0.31 (0.46)	0.13 (0.34)	0.14 (0.35)	0.08 (0.27)	0.23 (0.42)	0.41 (0.49)
Germany	0.48 (0.50)	0.37 (0.48)	0.04 (0.20)	0.06 (0.23)	0.47 (0.50)	0.06 (0.23)	0.06 (0.24)	0.01 (0.10)	0.36 (0.48)	0.27 (0.44)
Ghana	0.22 (0.42)	0.30 (0.46)	0.12 (0.32)	0.25 (0.43)	0.51 (0.50)	0.39 (0.49)	0.10 (0.31)	0.13 (0.33)	0.31 (0.46)	0.34 (0.47)
Greece	0.48 (0.50)	0.19 (0.40)	0.07 (0.25)	0.04 (0.19)	0.64 (0.48)	0.04 (0.21)	0.15 (0.36)	0.02 (0.13)	0.54 (0.50)	0.17 (0.38)
Hungary	0.68 (0.47)	0.33 (0.47)	0.13 (0.34)	0.05 (0.21)	0.58 (0.49)	0.03 (0.17)	0.19 (0.39)	0.03 (0.17)	0.56 (0.50)	0.25 (0.43)
Iceland	0.49 (0.50)	0.23 (0.42)	0.03 (0.17)	0.03 (0.18)	0.38 (0.48)	0.04 (0.19)	0.09 (0.29)	0.01 (0.08)	0.43 (0.50)	0.18 (0.38)
India	0.46 (0.50)	0.54 (0.50)	0.11 (0.31)	0.16 (0.37)	0.53 (0.50)	0.31 (0.46)	0.14 (0.34)	0.13 (0.33)	0.38 (0.49)	0.32 (0.47)
Indonesia	0.56 (0.50)	0.08 (0.26)	0.12 (0.33)	0.06 (0.23)	0.68 (0.47)	0.07 (0.26)	0.25 (0.43)	0.03 (0.17)	0.50 (0.50)	0.12 (0.33)
Iran	0.33 (0.47)	0.28 (0.45)	0.03 (0.16)	0.01 (0.12)	0.81 (0.39)	0.47 (0.50)	0.11 (0.31)	0.08 (0.27)	0.52 (0.50)	0.59 (0.49)
Ireland	0.50 (0.50)	0.57 (0.50)	0.04 (0.20)	0.07 (0.25)	0.69 (0.46)	0.25 (0.43)	0.11 (0.32)	0.05 (0.22)	0.41 (0.49)	0.36 (0.48)

Israel	0.55	0.39	0.13	0.14	0.55	0.15	0.10	0.05	0.50	0.35
	(0.50)	(0.49)	(0.34)	(0.35)	(0.50)	(0.35)	(0.31)	(0.23)	(0.50)	(0.48)
Italy	0.32	0.43	0.03	0.09	0.67	0.15	0.05	0.03	0.59	0.44
	(0.47)	(0.49)	(0.17)	(0.28)	(0.47)	(0.36)	(0.23)	(0.16)	(0.49)	(0.50)
Ivory Coast	0.26	0.39	0.19	0.21	0.30	0.29	0.21	0.36	0.34	0.37
	(0.44)	(0.49)	(0.39)	(0.41)	(0.46)	(0.45)	(0.41)	(0.48)	(0.47)	(0.48)
Japan	0.48	0.44	0.13	0.20	0.45	0.62	0.13	0.22	0.20	0.38
	(0.50)	(0.50)	(0.33)	(0.40)	(0.50)	(0.49)	(0.34)	(0.41)	(0.40)	(0.49)
Kazakhstan	0.42	0.44	0.06	0.13	0.45	0.24	0.05	0.07	0.18	0.30
	(0.49)	(0.50)	(0.23)	(0.34)	(0.50)	(0.42)	(0.22)	(0.26)	(0.39)	(0.46)
Kenya	0.44	0.41	0.14	0.14	0.51	0.30	0.15	0.13	0.40	0.37
	(0.50)	(0.49)	(0.34)	(0.35)	(0.50)	(0.46)	(0.36)	(0.33)	(0.49)	(0.48)
Latvia	0.36	0.25	0.11	0.08	0.24	0.04	0.10	0.03	0.29	0.16
	(0.48)	(0.43)	(0.31)	(0.27)	(0.42)	(0.19)	(0.29)	(0.18)	(0.46)	(0.37)
Malaysia	0.41	0.35	0.14	0.12	0.63	0.29	0.20	0.14	0.40	0.38
	(0.49)	(0.48)	(0.34)	(0.32)	(0.48)	(0.46)	(0.40)	(0.35)	(0.49)	(0.49)
Mexico	0.36	0.03	0.04	0.01	0.64	0.05	0.08	0.00	0.42	0.11
	(0.48)	(0.17)	(0.20)	(0.08)	(0.48)	(0.22)	(0.27)	(0.00)	(0.49)	(0.32)
Mozambique	0.38	0.14	0.19	0.04	0.40	0.07	0.18	0.04	0.32	0.11
	(0.48)	(0.35)	(0.39)	(0.19)	(0.49)	(0.26)	(0.38)	(0.20)	(0.47)	(0.31)
Netherlands	0.58	0.63	0.04	0.08	0.58	0.23	0.09	0.05	0.37	0.33
	(0.49)	(0.48)	(0.21)	(0.27)	(0.49)	(0.42)	(0.28)	(0.22)	(0.48)	(0.47)
Nigeria	0.37	0.31	0.22	0.21	0.58	0.40	0.17	0.17	0.38	0.27
	(0.48)	(0.46)	(0.41)	(0.41)	(0.49)	(0.49)	(0.37)	(0.38)	(0.49)	(0.45)
Peru	0.49	0.07	0.14	0.04	0.68	0.02	0.16	0.01	0.52	0.15
	(0.50)	(0.26)	(0.35)	(0.20)	(0.46)	(0.14)	(0.36)	(0.09)	(0.50)	(0.36)
Poland	0.41	0.63	0.06	0.35	0.40	0.40	0.08	0.17	0.22	0.49
	(0.49)	(0.48)	(0.24)	(0.48)	(0.49)	(0.49)	(0.27)	(0.38)	(0.41)	(0.50)
Portugal	0.51	0.17	0.06	0.02	0.68	0.03	0.09	0.01	0.59	0.15
	(0.50)	(0.38)	(0.23)	(0.15)	(0.47)	(0.17)	(0.29)	(0.08)	(0.49)	(0.36)
Russia	0.26	0.32	0.03	0.13	0.46	0.18	0.03	0.03	0.24	0.26
	(0.44)	(0.47)	(0.18)	(0.34)	(0.50)	(0.38)	(0.16)	(0.18)	(0.43)	(0.44)
Saudi Arabia	0.43	0.28	0.16	0.12	0.39	0.23	0.15	0.09	0.36	0.16
	(0.50)	(0.45)	(0.37)	(0.33)	(0.49)	(0.42)	(0.35)	(0.29)	(0.48)	(0.36)
Singapore	0.51	0.60	0.09	0.15	0.68	0.34	0.18	0.16	0.40	0.50
	(0.50)	(0.49)	(0.29)	(0.36)	(0.47)	(0.48)	(0.39)	(0.37)	(0.49)	(0.50)
Slovakia	0.46	0.57	0.14	0.39	0.40	0.28	0.12	0.17	0.46	0.54
	(0.50)	(0.50)	(0.35)	(0.49)	(0.49)	(0.45)	(0.32)	(0.37)	(0.50)	(0.50)
South Korea	0.56	0.28	0.15	0.02	0.85	0.22	0.42	0.07	0.58	0.25
	(0.50)	(0.45)	(0.36)	(0.16)	(0.36)	(0.42)	(0.49)	(0.25)	(0.49)	(0.43)
Spain	0.58	0.42	0.08	0.10	0.76	0.21	0.08	0.04	0.43	0.35
	(0.49)	(0.49)	(0.28)	(0.30)	(0.43)	(0.41)	(0.27)	(0.20)	(0.50)	(0.48)
Sri Lanka	0.28	0.49	0.05	0.12	0.66	0.47	0.09	0.05	0.42	0.56
	(0.45)	(0.50)	(0.23)	(0.32)	(0.47)	(0.50)	(0.28)	(0.22)	(0.49)	(0.50)
Sweden	0.53	0.32	0.11	0.06	0.57	0.05	0.16	0.00	0.41	0.21
	(0.50)	(0.46)	(0.31)	(0.24)	(0.50)	(0.23)	(0.37)	(0.07)	(0.49)	(0.41)
Thailand	0.20	0.05	0.05	0.03	0.55	0.12	0.12	0.02	0.32	0.22
	(0.40)	(0.23)	(0.22)	(0.17)	(0.50)	(0.32)	(0.32)	(0.16)	(0.47)	(0.41)
Trinidad and	0.41	0.56	0.11	0.20	0.50	0.40	0.15	0.16	0.32	0.44
Tobago	(0.49)	(0.50)	(0.31)	(0.40)	(0.50)	(0.49)	(0.36)	(0.37)	(0.47)	(0.50)
Turkey	0.45	0.25	0.02	0.02	0.71	0.15	0.11	0.04	0.52	0.21
	(0.50)	(0.43)	(0.13)	(0.13)	(0.45)	(0.36)	(0.32)	(0.19)	(0.50)	(0.40)
UAE	0.57	0.56	0.05	0.07	0.60	0.21	0.12	0.03	0.41	0.31
	(0.49)	(0.50)	(0.21)	(0.25)	(0.49)	(0.41)	(0.32)	(0.18)	(0.49)	(0.46)
Ukraine	0.30	0.39	0.02	0.13	0.30	0.17	0.02	0.03	0.31	0.35
	(0.46)	(0.49)	(0.13)	(0.34)	(0.46)	(0.37)	(0.13)	(0.17)	(0.46)	(0.48)
United	0.54	0.67	0.06	0.11	0.51	0.14	0.12	0.05	0.32	0.27
Kingdom	(0.50)	(0.47)	(0.24)	(0.32)	(0.50)	(0.35)	(0.33)	(0.23)	(0.47)	(0.45)
United States	0.62	0.63	0.09	0.16	0.54	0.16	0.10	0.05	0.41	0.30
	(0.48)	(0.48)	(0.29)	(0.37)	(0.50)	(0.37)	(0.30)	(0.22)	(0.49)	(0.46)

Vietnam	0.50 (0.50)	0.10 (0.30)	0.09 (0.29)	0.03 (0.17)	0.53 (0.50)	0.02 (0.14)	0.09 (0.28)	0.01 (0.08)	0.32 (0.47)	0.06 (0.24)

Supplementary Table S6. Average number of emotions selected per country.

Country	Average number of emotions selected
Algeria	1.28
Argentina	1.50
Armenia	1.63
Australia	1.81
Austria	1.65
Bosnia and Herzegovina	1.66
Botswana	1.68
Brazil	1.73
Canada	1.86
Chile	1.70
China	1.73
Colombia	1.70
Czech Republic	1.78
Ecuador	1.62
Estonia	1.67
Finland	1.66
Germany	1.67
Ghana	1.69
Greece	1.55
Hungary	1.88
Iceland	1.72
India	1.66
Indonesia	1.77
Iran	1.84
Ireland	1.64
Israel	1.75
Italy	1.66
Ivory Coast	1.96
Japan	2.03
Kazakhstan	1.56
Kenya	1.79
Latvia	1.47
Malaysia	1.86
Mexico	1.46
Mozambique	1.88
Netherlands	1.75
Nigeria	1.80
Peru	1.48
Poland	1.74
Portugal	1.60
Russia	1.55
Saudi Arabia	1.46
Singapore	1.91
Slovakia	1.75
South Korea	1.83
Spain	1.83
Sri Lanka	1.68
Sweden	1.86

Thailand	1.57
Trinidad and Tobago	1.69
Turkey	1.78
UAE	1.80
Ukraine	1.57
United Kingdom	1.68
United States	1.76
Vietnam	1.66

Listening to music on headphones:

Country	Average number of emotions selected
Algeria	1.62
Argentina	1.66
Armenia	1.82
Australia	2.34
Austria	2.20
Bosnia and Herzegovina	2.29
Botswana	2.24
Brazil	1.89
Canada	2.59
Chile	2.00
China	2.06
Colombia	1.89
Czech Republic	2.31
Ecuador	1.71
Estonia	1.87
Finland	1.95
Germany	2.16
Ghana	1.71
Greece	1.81
Hungary	2.49
Iceland	2.17
India	2.14
Indonesia	2.01
Iran	1.81
Ireland	2.08
Israel	2.21
Italy	1.93
Ivory Coast	1.95
Japan	2.70
Kazakhstan	1.93
Kenya	2.03
Latvia	1.71
Malaysia	2.01
Mexico	1.51
Mozambique	2.13
Netherlands	2.39
Nigeria	1.84
Peru	1.67
Poland	2.12
Portugal	1.99

Russia	1.70
Saudi Arabia	1.80
Singapore	2.25
Slovakia	2.16
South Korea	2.10
Spain	2.43
Sri Lanka	1.84
Sweden	2.35
Thailand	1.54
Trinidad and Tobago	1.88
Turkey	2.00
UAE	2.40
Ukraine	1.79
United Kingdom	2.34
United States	2.43
Vietnam	1.91

Sleeping in a restaurant:

Country	Average number of emotions selected
Algeria	1.21
Argentina	1.27
Armenia	1.37
Australia	1.42
Austria	1.39
Bosnia and Herzegovina	1.35
Botswana	1.47
Brazil	1.38
Canada	1.48
Chile	1.36
China	1.21
Colombia	1.32
Czech Republic	1.46
Ecuador	1.35
Estonia	1.52
Finland	1.45
Germany	1.35
Ghana	1.55
Greece	1.30
Hungary	1.47
Iceland	1.38
India	1.36
Indonesia	1.42
Iran	1.33
Ireland	1.21
Israel	1.47
Italy	1.34
Ivory Coast	1.79
Japan	1.46
Kazakhstan	1.30
Kenya	1.46

Latvia	1.43
Malaysia	1.51
Mexico	1.25
Mozambique	1.69
Netherlands	1.31
Nigeria	1.68
Peru	1.25
Poland	1.56
Portugal	1.18
Russia	1.38
Saudi Arabia	1.36
Singapore	1.44
Slovakia	1.57
South Korea	1.35
Spain	1.46
Sri Lanka	1.31
Sweden	1.65
Thailand	1.31
Trinidad and Tobago	1.43
Turkey	1.34
UAE	1.36
Ukraine	1.40
United Kingdom	1.33
United States	1.42
Vietnam	1.50

Singing in a library:

Country	Average number of emotions selected
Algeria	1.39
Argentina	1.51
Armenia	1.78
Australia	1.77
Austria	1.58
Bosnia and Herzegovina	1.56
Botswana	1.73
Brazil	1.86
Canada	1.82
Chile	1.78
China	2.11
Colombia	1.83
Czech Republic	1.79
Ecuador	1.72
Estonia	1.50
Finland	1.74
Germany	1.70
Ghana	1.94
Greece	1.57
Hungary	1.79
Iceland	1.69
India	1.60

Indonesia	1.76
Iran	2.37
Ireland	1.77
Israel	1.71
Italy	1.68
Ivory Coast	2.05
Japan	2.37
Kazakhstan	1.68
Kenya	1.88
Latvia	1.42
Malaysia	2.00
Mexico	1.64
Mozambique	2.05
Netherlands	1.84
Nigeria	2.06
Peru	1.53
Poland	1.86
Portugal	1.59
Russia	1.73
Saudi Arabia	1.50
Singapore	2.03
Slovakia	1.70
South Korea	2.13
Spain	1.98
Sri Lanka	1.97
Sweden	1.81
Thailand	1.81
Trinidad and Tobago	1.80
Turkey	2.11
UAE	1.84
Ukraine	1.65
United Kingdom	1.67
United States	1.69
Vietnam	1.70

Reading a newspaper at the movies:

Country	Average number of emotions selected
Algeria	1.07
Argentina	1.23
Armenia	1.27
Australia	1.36
Austria	1.20
Bosnia and Herzegovina	1.17
Botswana	1.20
Brazil	1.41
Canada	1.31
Chile	1.27
China	1.17
Colombia	1.27
Czech Republic	1.26

Ecuador	1.29
Estonia	1.66
Finland	1.31
Germany	1.26
Ghana	1.41
Greece	1.23
Hungary	1.37
Iceland	1.34
India	1.31
Indonesia	1.53
Iran	1.35
Ireland	1.25
Israel	1.29
Italy	1.18
Ivory Coast	1.79
Japan	1.54
Kazakhstan	1.18
Kenya	1.50
Latvia	1.28
Malaysia	1.67
Mexico	1.17
Mozambique	1.64
Netherlands	1.26
Nigeria	1.50
Peru	1.21
Poland	1.42
Portugal	1.16
Russia	1.17
Saudi Arabia	1.28
Singapore	1.51
Slovakia	1.31
South Korea	1.56
Spain	1.21
Sri Lanka	1.24
Sweden	1.43
Thailand	1.32
Trinidad and Tobago	1.43
Turkey	1.38
UAE	1.34
Ukraine	1.19
United Kingdom	1.31
United States	1.28
Vietnam	1.36

Violating a cooperation norm:

**Country**	**Average number of emotions selected**
Algeria	1.14
Argentina	1.86
Armenia	1.96
Australia	2.15

Austria	1.88
Bosnia and Herzegovina	1.94
Botswana	1.78
Brazil	2.13
Canada	2.08
Chile	2.06
China	2.16
Colombia	2.20
Czech Republic	2.06
Ecuador	2.03
Estonia	1.81
Finland	1.88
Germany	1.89
Ghana	1.83
Greece	1.86
Hungary	2.31
Iceland	2.02
India	1.90
Indonesia	2.14
Iran	2.34
Ireland	1.89
Israel	2.09
Italy	2.15
Ivory Coast	2.23
Japan	2.09
Kazakhstan	1.73
Kenya	2.10
Latvia	1.52
Malaysia	2.14
Mexico	1.71
Mozambique	1.91
Netherlands	1.93
Nigeria	1.95
Peru	1.76
Poland	1.76
Portugal	2.10
Russia	1.77
Saudi Arabia	1.38
Singapore	2.29
Slovakia	2.04
South Korea	1.99
Spain	2.08
Sri Lanka	2.04
Sweden	2.03
Thailand	1.86
Trinidad and Tobago	1.90
Turkey	2.03
UAE	2.05
Ukraine	1.80
United Kingdom	1.73
United States	1.98

Vietnam	1.86

Supplementary Table S7. Correlations between autonomy and other variables

Predictor	Correlation with Autonomy
Indulgence	-0.16 [-0.43, 0.13]
Power distance	-0.47 [-0.66, -0.21]
Individualism	0.40 [0.13, 0.61]
Tightness	-0.48 [-0.66, -0.25]
Median per-capita income	0.42 [0.16, 0.63]
Gender equality	0.41 [0.17, 0.61]

Note. Correlations are based on n = 56 countries, except for indulgence (n = 48), power distance (n = 50), individualism (n = 50), and median income (n = 49). Measures of indulgence, power distance, and individualism are from Hofstede et al.. Autonomy and tightness was measured in the current study. gender equality is measured by the Global Gender Gap Index from World Economic Forum ; median per-capita income measures are from Gallup.

Supplementary Figure SF2. Model depicted in the main manuscript as Figure 3, here with Tightness replacing Autonomy.

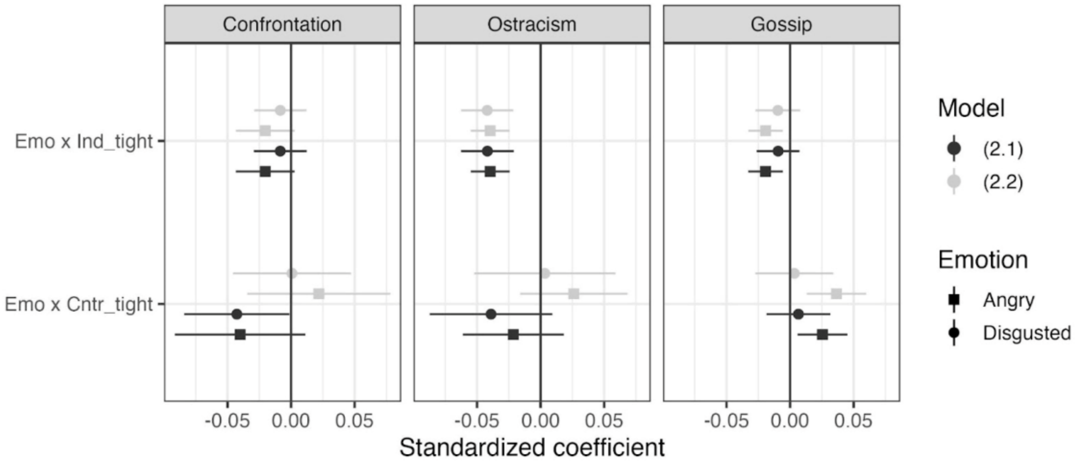

Supplementary Figure SF3. Model depicted in the main manuscript as Figure 1, here including the ratings of appropriateness in the same model.

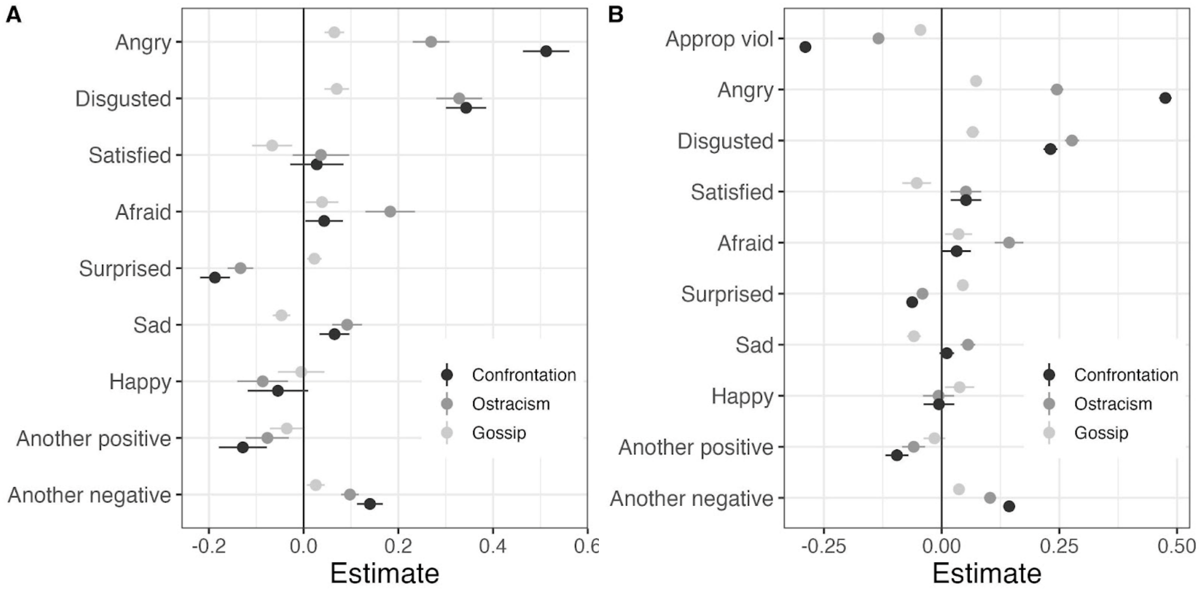

Supplement: Supplementary file 1 — Supplementary Information. [file 41598_2024_55815_MOESM1_ESM.pdf]
